# Defense related decadienal elicits membrane lipid remodeling in the diatom *Phaeodactylum tricornutum*

**DOI:** 10.1371/journal.pone.0178761

**Published:** 2017-06-05

**Authors:** Tanya Sabharwal, Kanagasabapathi Sathasivan, Mona C. Mehdy

**Affiliations:** Department of Molecular Biosciences, University of Texas at Austin, Austin, Texas, United States of America; Stazione Zoologica Anton Dohrn, ITALY

## Abstract

Diatoms rapidly release extracellular oxylipins (oxygenated lipids) including polyunsaturated aldehydes in response to herbivory and other stresses. Oxylipins have several defense-related activities including inhibition of reproduction in herbivores and signaling to distant diatoms. Physiological changes in diatoms exposed to varying levels of oxylipins are only beginning to be understood. In this study, *Phaeodactylum tricornutum* cultures were treated with sublethal concentrations of the polyunsaturated aldehyde *trans*,*trans*-2,4-decadienal (DD) to assess effects on lipid composition and membrane permeability. In cells treated with DD for 3 hr, all measured saturated and unsaturated fatty acids significantly decreased (0.46–0.69 fold of levels in solvent control cells) except for 18:2 (decreased but not significantly). The decrease was greater in the polyunsaturated fatty acid pool than the saturated and monounsaturated fatty acid pool. Analysis of lipid classes revealed increased abundances of phosphatidylethanolamine and phosphatidylcholine at 3 and 6 hr. Concomitantly, these and other membrane lipids exhibited increased saturated and monounsaturated acyl chains content relative to polyunsaturated acyl chains compared to control cells. Evidence of decreased plasma membrane permeability in DD treated cells was obtained, based on reduced uptake of two of three dyes relative to control cells. Additionally, cells pre-conditioned with a sublethal DD dose for 3 hr then treated with a lethal DD dose for 2 hr exhibited greater membrane integrity than solvent pre-conditioned control cells that were similarly treated. Taken together, the data are supportive of the hypothesis that membrane remodeling induced by sublethal DD is a key element in the development of cellular resistance in diatoms to varying and potentially toxic levels of polyunsaturated aldehydes in environments impacted by herbivory or other stresses.

## Introduction

Microalgae are the most efficient photosynthetic organisms on earth [1]. Apart from their central ecological importance, specific microalgae species have been identified as potential sources of commercial products including biofuels and nutritional oils due to their diverse and abundant lipid content [2,3] and bioactive metabolites including potential drugs [4, 5]. However, reliable and high yields of biofuels and other products have generally not been fully realized in large-scale cultivation. Major factors limiting biomass and product yields are exposures to abiotic stresses such as high light, high temperature, nutrient deprivation [6,7] and biotic stresses such as herbivory by copepods and other invertebrates [8,9]. To enhance resistance to herbivory by diverse grazers and better understand ecological relationships, it is important to study defense mechanisms that have evolved in microalgae.

Within phytoplankton groups rich in lipids, marine diatoms have been recognized as a key foundational group in food webs supporting aquaculture, commercial fisheries, and ecosystems. Diatoms generally serve as important food sources for copepods and juvenile invertebrates either singly or in mixed populations with other microalgal species [10, 11]. However, many species have potent mechanisms to deter grazers and their molecular defense mechanisms have been relatively well studied [12–16]. In response to cell damage by grazers or mechanical cell lysis (sonication) [17–22] and abiotic stresses such as aging and nutrient limitation [23,24], diatoms rapidly release bioactive compounds called oxylipins into the surrounding seawater. These diatom species-specific diverse oxylipins include polyunsaturated aldehydes (PUAs) and oxo-acids. Membrane lipids are hydrolyzed into free polyunsaturated fatty acids (PUFAs), which are then acted on by enzymes such as lipoxygenases and hyroperoxide lyases to generate PUAs [14, 19, 21, 25]. Oxylipins have been shown to inhibit reproductive ability and normal development of grazers such as copepods and other invertebrates [26–28]. Depending on the interaction, after exposure to diatom-derived oxylipins, grazers exhibited cell cycle disruptions, apoptosis and increased expression of genes associated with antioxidant defenses such as aldehyde dehydrogenase, catalase, and glutathione synthase [29, 30, 31] and altered swimming behavior [32]. In addition, oxylipins from the damaged and dying diatoms elicit physiological effects in distant ungrazed diatoms, which may improve their survival in a zone of active herbivory [13, 31]. These effects include a rapid increase in intracellular Ca^2+^, nitric oxide (NO) levels, and increased photosystem protective defenses [13, 33].

One well-studied model PUA is *trans*,*trans*-2,4-decadienal (DD) that has various physiological effects on ungrazed diatoms [13, 34–36]. In laboratory settings, the marine diatom *Phaeodactylum tricornutum* has been particularly employed for molecular studies due to ease of growth, sequenced genome, ability to be transformed [37–39], and defense responses involving production of non-volatile aldehydic acids such as oxo-acids 12-oxo-(5Z,8Z,10E)-dodecatrienoic acid (12-ODTE) and 9-oxo-(5Z,7E)-nonadienoic acid (9-ONDE) that inhibit normal development of invertebrates [17, 40]. Vardi et al. [13] showed that DD treatment of unstressed *P*. *tricornutum* cells resulted in calcium-dependent NO production within 5 min and genome-wide transcriptome changes measured within 6 hr. Cell populations treated with DD (3.3 µM-to 13.2 µM) for 24 hr when mixed with a population of cells previously unexposed to DD, induced NO production in the unexposed cells. This indicated transmission of a DD induced diffusible signal from exposed to unexposed cells but the nature of the signal and mechanism were not elucidated. Of particular relevance to this study, cells that were pretreated with sublethal levels of DD for 2 hr and then exposed to lethal levels had increased resistance to subsequent exposure to lethal levels, increased growth and photosynthetic efficiency. Taken together, these results suggested a physiological defense related benefit, which may enable cells to survive in areas of intense herbivory [13,41]. However, the mechanism of action for developing improved DD resistance in cells pretreated with sublethal DD is unknown.

This study explored lipid composition and membrane function in healthy *P*. *tricornutum* cells treated with sublethal DD concentration within a short time scale (3 and 6 hr). We hypothesized that DD exposure in distant *P*. *tricornutum* cells in zones of active herbivory may induce rapid changes in membrane lipids as a part of the stress response and hence alter plasma membrane permeability to reduce uptake of DD which may vary widely in concentration in the zone of herbivory. We postulated that this is an adaptive response to maintain viability. There are many studies demonstrating altered lipid compositions in various unicellular algae in response to abiotic stresses such as high light, high temperature, nutrient limitation, exposure to heavy metals etc [7] and in higher plants in response to wounding [42]. In the eustigmatophyte alga *Nannochloropsis oculata*, DD treatment for 24 hr significantly increased neutral lipids [43]. However, the effects of DD on membrane lipids in microalgae species including diatoms are currently unknown and was addressed in this study along with the effects on other lipid pools.

## Materials and methods

### Strain and culture conditions

**Fig 1:**
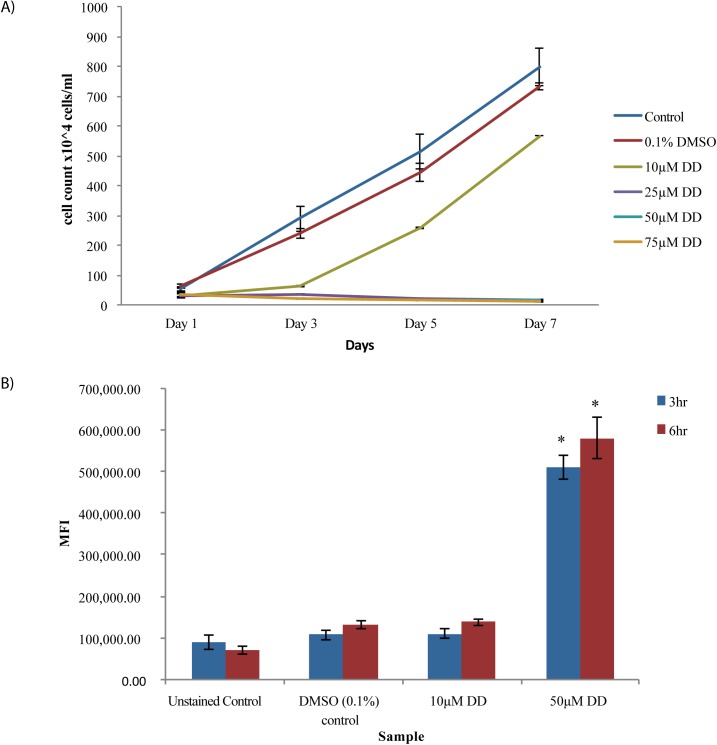
Effects of DD concentration on cell growth and viability. (A) Cell densities were measured with a hemocytometer. Error bars represent standard error. Significance calculated using student’s t-test, P< 0.05 for 25 µM, 50 µM and 75 µM DD with respect to DMSO solvent control and P> 0.05 for 10µM DD with respect to DMSO solvent control; n = 3. Representative data from three independent experiments are shown. (B) Measurement of viability in cells treated with DMSO and DD dose for the times indicated (hr). Cell accumulation of Sytox Green fluorescent dye was measured by flow cytometry. MFI is Median Fluorescence Intensity. Error bars represent standard error. Changes with 50 µM DD were found significant with respect to DMSO solvent control, student’s T-test, * p<0.05; n = 3.

*Phaeodactylum tricornutum* (CCMP2561) from Provasoli-Guillard National Center for Culture of Marine Phytoplankton (CCMP- http://serc.carleton.edu/resources/20043.html), USA was grown in f/2 medium made with steam-sterilized local seawater with f/2 vitamins (filter sterilized) and inorganic nutrients. Cultures were incubated under cool-white fluorescent lights at 80 μmol m^−2^ s^−1^ on a 12:12 hr light/dark cycle at 20°C for about 10–12 days and swirled by hand every 2–3 days. Cells were induced with decadienal or DMSO solvent treatments at 3 hr into light cycle (see figure legends for concentrations used). Cells were grown to log phase (2.5-3x10^6^ cells/ml) for most experiments except experiments presented in Fig 1A, S1 and S2 Figs used the cell densities listed in their legends. Biological replicates were cultures grown in independent flasks. Cells were harvested by centrifugation for 10 min at 3500g.

### Chemicals

Chemicals and vitamin stocks for preparing f/2 media were obtained from UTEX culture collection, University of Teas at Austin. SNP (sodium nitroprusside; 100 mM fresh stock in water), anhydrous DMSO and *trans*,*trans*-2,4-decadienal (DD, W313505), Evans blue (E2129-10G) were obtained from Sigma-Aldrich. DD was freshly prepared as 10 mM stock in DMSO. DAF-FM-Diacetate (powder, D23844), Sytox Green nucleic acid stain (5 mM stock in DMSO; S7020), BODIPY 505/515 (10 mg powder; D3921), Hoechst 33342 (10 mg/ml in water; H3570) and CFDA-SE (5-(and-6)-Carboxyfluorescein Diacetate, Succinimidyl Ester, powder, C34554) were purchased from Thermo Fisher Scientific, Molecular Probes division. Tri-Tri Decanoin (13:0 internal standard) was purchased from Nu Chek Prep, Inc. All chemicals and solvents used for fatty acid methyl ester preparation and total lipid extraction were either ACS or HPLC grade purchased from Fisher Scientific.

### Growth assay

Cells (initial density 5x10^5^ cells/ml) were either incubated with DD or DMSO (0.1%) solvent control and grown over a period of 7 days into late log phase. Cells were observed under microscope for normal morphology of cells and counts were done on alternate days using a hemocytometer (Reichert Co.) to determine growth of cells.

### Viability assay

Cells were treated with either water control, DMSO (0.1%) solvent control or decadienal (10 µM and 50 µM). Cells (50 ml) were harvested at the indicated times (hr), resuspended in 10 mM Hepes buffer, pH 7.3, Sytox Green added to 1 µM and incubated for 10 min according to manufacturer’s protocol. Cells were assayed on BD LSR Fortessa flow cytometer.

### Measurement of NO production in cells

Cells were treated with either water control, DMSO (0.1%) solvent control or decadienal (10 µM and 50 µM). NO was measured according to Vardi et al, 2006 [13] with modifications. Cells (50 ml) were harvested at the indicated times (hr) and resuspended in 10 μM DAF-FM-Diacetate in 10 mM Hepes pH 7.3 buffer. After incubation in the dark for 60 min, cells were twice centrifuged and washed in 10 mM Hepes with 30 min incubation after the first wash to allow deesterification. Efficiency of loading was tested by examining DAF-FM–dependent fluorescence in the microscope following addition of the NO donor SNP (0.5 mM) to cells. To quantify NO accumulation, DAF-FM fluorescence was measured using a BD LSR Fortessa flow cytometer equipped with a 488 nm laser as excitation source. A 530/30BP emission filter was used for detection of DAF-FM–derived fluorescence.

### FAME preparation and analysis

Fatty acid methyl esters (FAMEs) were prepared directly from cell pellets using a protocol modified from Zhou et al. [44]. Five biological replicates of DMSO (0.1%) solvent control and 6–8 biological replicates of 10 µM DD treated samples were prepared for each time point (3 and 6 hr). Cells (~1–1.5x10^8^) were harvested, cell pellets were flash frozen with liquid nitrogen then lyophilized for 3–4 hr and frozen in -80°C until use. Internal standard 13:0 (Tri Tri Decanoin) was added to each sample before starting FAME preparation. The pellets were suspended in 2–3 ml 100% methanol to dissolve and then transferred to Kimax glass (45066A-16125) tubes for FAME preparation. To minimize losses due to oxidation of fatty acids, solvents were degassed by bubbling N_2_ gas, BHT (0.01%) was added to solvents, and N_2_ gas was blown intermittently on samples during preparation. After FAME preparation, fixed volume of each sample (processed upper phase) was transferred to 2 ml capped glass vials (ThermoFisher 60180–599), completely dried under nitrogen gas and sent on dry ice for GC-MS analysis at the University of Texas Health Science Center at San Antonio Mass Spectrometry Laboratory.

### GC/MS analyses of FAMES

Gas chromatography/mass spectrometry (GC/MS) analyses were performed on a ThermoFisher (San Jose, CA) TRACE DSQ single quadrupole mass spectrometer at Mass Spectrometry Lab, The University of Texas at San Antonio (http://research.uthscsa.edu/mass-spectrometry/). GC conditions were as follows: column, DB-225MS (Agilent; Santa Clara, CA), 30 m x 0.25 mm, 25 µm film thickness; carrier gas, helium; linear velocity, 1 ml/min (constant flow); injection volume, 1 µl; injection, splitless, 50 ml/min split flow after 1 min; injector temperature, 220°C; column temperature program, initial temperature of 50°C held for 1 min followed by an increase to 200°C at 50°C/min then an increase to 240°C at 10°C/min. MS conditions were: ionization, EI (70 eV); detection, positive ion; full scan analyses, m/z 50 –m/z 400 at two scans/sec. Peak areas were obtained by manual integration of total ion current chromatograms, using Xcalibur (ThermoFisher). Each fatty acid peak in the GC chromatogram was identified based on the GLC-85 mixture reference standard (Nu Chek Prep, Inc.). Area was determined for each peak and relative proportion for each peak was determined by dividing the area of the peak by the area of internal standard. Fold changes in specific fatty acid were determined by dividing the relative proportion of certain fatty acid in DD treated samples with relative proportion of that fatty acid in DMSO solvent control.

### Lipid molecular species preparation and ESI-MS/MS analysis

Total lipids were extracted from cell pellets using a procedure modified from Welti et al. [45]. Five biological replicates of each of DMSO (0.1%) solvent control and 10 µM DD treated cells were prepared for each time point (3 and 6 hr). Cells (~1.4x10^8^) were harvested, cell pellet was flash frozen with liquid nitrogen, lyophilized and then stored in -80^°^C until lipid extraction. To the lyophilized pellet, 2 ml of chloroform and 4 ml of methanol were added, tubes were vortexed to mix and then 2 ml chloroform and 2 ml water were added. Tubes were vortexed and then centrifuged at 3500 rpm for 10 min. Lower phase was collected in a clean glass tube. Two ml chloroform was added, vortexed and centrifuged and lower phase was collected. Lower phases were combined and washed once with 0.5 ml 1 M KCl and once with 0.5 ml water. Extracted lipids samples were dried under nitrogen and sent for electron spray ionization-mass spectrometry and tandem mass spectrometry (ESI-MS/MS) analysis at the Analytical Laboratory of the Kansas Lipidomics Research Center (https://www.k-state.edu/lipid/).

### Sterol extraction and GC-MS analysis

Total lipids were extracted from 1.5x10^8^ cells from 4 replicates of DMSO solvent control cells and 5 replicates of 10 µM DD treated cells at 3 hr and 6 hr using the protocol as described earlier (modified from Welti et al.) [45]. Extracted lipids were completely dried under nitrogen gas and shipped to Kansas State University (KSU) lipidomics center where samples were further processed. Each sample was re-dissolved in 1 ml chloroform and 700 µl was used for saponification. 4 µl of internal standard-cholestanol (Stock solution: 1 mg in 1 ml of chloroform, Steraloids, Inc) was added to samples and samples dried under nitrogen. Then, 1 ml of methanolic potassium hydroxide (3 M KOH: MeOH v/v 1:9) was added and heated at 80°C for 1 hr. After cooling down, 2 ml of HPLC-grade water was added and the sterols were recovered by extracting the mixture 3 times with 2 ml hexane. The hexane extracts were pooled and dried under nitrogen. Each dried extract was re-dissolved in 70 µl pyridine and 30 µl N-trimethylsilyl-N-methyltrifluoroacetamide with trimethylchlorosilane was added and incubated at 50°C for 60 min. Each sample extract was dried completely under nitrogen and re-dissolved in 100 µl hexane. For analysis, 1 µl of each sample was injected to GC-MS.

GC-MS was performed on an Agilent 6890N GC coupled to an Agilent 5975N quadrupole mass selective detector. The GC was fitted with a DB-5MS capillary column with a 5% phenyl, 95% methylpolysiloxane stationary phase (column length: 60 m, internal diameter: 250 µm, film thickness: 0.25 µm). Helium was used as the carrier gas at a column flow rate of 1 ml/min. The front inlet was operating at a pressure of 24.93 psi and 280°C. The Agilent 7683 autosampler was used to inject 3 µl of the sample in the splitless mode. The GC oven temperature program was: initial temperature of 150°C, hold 1 min. increase 30°C/min to 300°C, then increase 3°C/min to a final temperature of 315°C, hold 5 min. Total run time was 16.00 min. The mass spectrometer was operated in the electron impact mode at 70 eV ionization energy. The MS quad temperature was at 150°C and the MS source temperature was at 230°C. The data acquisition was at scan mode. Scan mass was 50 to 650. The data were processed with Agilent Chemstation software.

### Measurement of neutral lipids by Bodipy fluorescent dye

For intracellular neutral lipids determination, the protocol for staining cells with Bodipy was adapted and modified from Govender et al. [46] and that recommended by manufacturer. The fluorescent dye, BODIPY 505/515, was prepared in anhydrous DMSO as 0.5 mg/ml stock solution. Three biological replicates of each of DMSO (0.1%) solvent control and 10 µM DD treated cells were prepared for each time point (3 and 6 hr). Cells (~1.4x10^8^) were harvested, resuspended in 10 mM Hepes buffer pH 7.3 with a final concentration of 0.075 µg/ml Bodipy dye and incubated for 10 min at room temperature. Cells were assayed within 30 min of dye addition on BD Accuri flow cytometer (equipped with 488 nm laser) to quantify lipid-based fluorescence.

### Analysis of volatiles

For rapid screening and analysis of volatiles, solid phase microextraction was performed with a polydimethylsiloxan fiber using the procedure modified from that described in Pohnert et al. (2002) [17]. Cells (50 ml, ~2.8x10^6^ cells/ml) of DMSO (0.1%) solvent control and 10 µM DD treated cultures (3 hr and 6 hr time points) were harvested, washed once in f/2 media then resuspended in 3 ml fresh f/2 media in glass (Supelco 2–7346) vials which were sealed using a Teflon cap. A polydimethylsiloxane-coated (100 µm; PDMS/DVB) fiber (Supleco- 57310U) was introduced in the headspace over the medium. Extraction was performed for 15 min at 50°C with continuous stirring of culture. Evaporation of the analytes from the fiber was directly performed within the injection port (220°C) of the GC-MS (DB225-MS column, Alltech; T-program: 50°C [2 min, splitless]; ramped with 10°C min–1 to 200°C and with 30°C min–1 to 280°C [2 min]). Decadienal standard (10 µM mixed in media alone) was also run as a control. Five independent experiments, each with five biological replicates of treated cells were analyzed.

### Membrane permeability assays

Cells were treated with DMSO (0.1%) or 10 µM DD for 3 hr and 6 hr. After these periods, dyes were applied in 3 independent experiment trials (each trial with 3–5 replicates for solvent control and DD treated cells at each time point). Staining of cells with Evans blue was done as described [47]. Using 1% stock solution, dye was added to 50 ml cells at final concentration of 0.02% and incubated for 15 min before counting on a hemocytometer. Counting was done in triplicates and average was taken for final count of stained cells. Data were expressed as % stained cells and statistical significance was calculated by two sample unpaired independent student’s t-test. Staining cells with Hoechst 33342 followed manufacturer’s (Molecular Probes) recommended procedure. Cells (50 ml) were harvested, resuspended in 10 mM Hepes buffer pH 7.3 and Hoechst 33342 was added to 5 µg/ml and incubated for 10 min in dark before assaying on a Zeiss Axiovert 200M fluorescent microscope and by BD FACSAria flow cytometer with DAPI filter set (Ex 350/ Em 461). Staining cells with CFDA-SE dye was performed as described in manufacturer’s protocol (Molecular Probes). Cells (50 ml) were harvested, resuspended in 10 mM Hepes buffer pH 7.3, CFDA-SE (5 mM in DMSO stock) was added at 5 µM final concentration and incubated for 20 min in dark at room temperature. Five times original staining volume of Hepes buffer was added to the cells and incubated for 5 min. Cells were washed by centrifugation for 5 min and resuspended in fresh buffer and incubated for 10 min before imaging at Zeiss Axiovert 200M fluorescent microscope and assaying on BD Acurri flow cytometer with FITC filter set (Ex 492/ Em 517). Microscope images were processed using Fiji (Image J) and flow cytometer data was analyzed for median fluorescence intensity (MFI) of solvent control and DD treated cells. Statistical significance was calculated using two sample unpaired independent student’s t-test.

### Membrane integrity assays of cells pretreated with sublethal DD followed by lethal DD concentration

Cells (80ml; density ~ 2.6x10^6^ cells/ml) were treated with either DMSO control (0.1%) or DD (10 µM) for 3 hr. Cells were harvested by centrifugation at 3500 rpm for 8 minutes and then resuspended in fresh F/2 media. The culture was then equally divided (20 ml each) into four 250 ml flasks and treated with either DMSO (0.1%) or DD (10 µM or 50 µM) for 2 hr. Cells were then harvested by centrifugation at 3500 rpm for 10 minutes and then resuspended in 10 mM Hepes buffer, pH 7.3. Sytox Green was added to 1 µM and incubated for 10 min according to manufacturer’s protocol. Cells were assayed on a BD LSR Fortessa flow cytometer. The experiment was done with three biological replicates and each biological replicates had three technical replicates for assaying fluorescence on flow cytometer except the data for 50 µM DD concentration represented 2 biological replicates.

## Results

### Growth and viability assay to determine effect of DD on cells

As different diatom cultures vary in growth, viability, and responsiveness to decadienal, it was important to establish a suitable sublethal DD concentration used in our *P*. *tricornutum* culture system. The goal was to identify a DD concentration low enough so that growth and viability were preserved while being high enough to induce a known response, an increase in intracellular NO [13]. Growth assay showed that 10 µM DD slowed growth relative to controls in the first 3 days but cells then resumed growth comparable to controls over the 7 day assay period (Fig 1A). In contrast, concentrations of DD at 25 µM and above caused severe growth inhibition throughout the assay period.

Cell viability was measured at the 3 and 6 hr time points chosen for sublethal DD exposure and at a cell density range that is feasible in scale for lipid measurements. Cell viability was assayed using Sytox Green staining and flow cytometry. Sytox Green is highly excluded from living cells while dead cells or cells with compromised membrane integrity take up the dye, which binds DNA and produces a highly fluorescent signal. Sytox Green has been used in previous studies of the effects of decadienal and other PUA on diatoms including *P*. *tricornutum* [13, 34]. In agreement with the modest effects of 10 µM DD on cell growth, Sytox Green fluorescence in log phase cells (2.5-3x10^6^ cells/ml) treated with 10 µM DD for 3 and 6 hr, was 1.02 fold and 1.05 fold, respectively, compared to DMSO (0.1%) solvent control (Fig 1B). In contrast, the fluorescence in cells treated with cell growth blocking 50 µM DD were 4.76 fold and 4.4 fold higher, respectively. Cells were also assessed at an earlier log phase (8-9x10^5^ cells/ml) to determine whether 10 µM DD was tolerated well at a different growth stage. The same comparisons at 6 hr were 1.2 fold for 10 µM DD treated cells and 13.4 fold for 50 µM DD (S1 Fig.). In summary, the growth and viability measurements demonstrated 10 µM as a potential workable DD concentration.

### DD concentration and time dependent NO levels in cells

**Fig 2:**
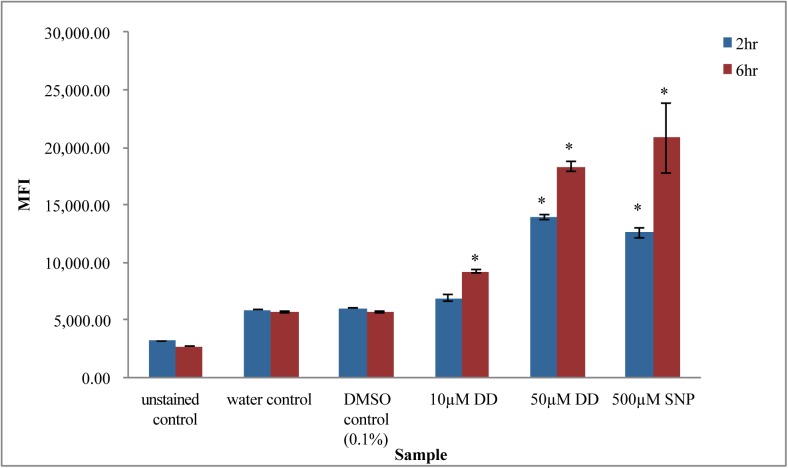
Effects of DD concentration and time on NO accumulation. NO accumulation in cells was measured using fluorescent dye DAF-FM diacetate by flow cytometry. MFI is Median Fluorescence Intensity. Unstained control and stained DMSO (0.1%) solvent control were used as negative controls. SNP (which is a NO donor) at 500 µM. acted as positive control. Error bars represent standard error. (*Significance with respect to DMSO solvent control calculated by student’s t-test, p<0.05, n = 3).

Previously, *P*. *tricornutum* cells treated with DD were shown to produce intracellular NO over several hours in a concentration and time dependent manner [13, 33]. In both studies, NO assay was accomplished by use of a cell permeable nonfluorescent dye widely used for intracellular NO measurements, DAF-FM diacetate. The acetate groups are removed by cellular esterases and DAF-FM reaction with NO produces a highly fluorescent product. NO-dependent fluorescence was supported by the use of NO scavenger, NO donor and NOS inhibitor controls employed in these studies, The DAF-FM diacetate assay was adopted in our experiments and NO dependence was validated for all time points by significantly higher fluorescence signal when cells were incubated with the NO donor, SNP (Fig 2, S2 Fig.). Cells at later and early log stages exhibited increased NO in a DD concentration and time dependent manner (Fig 2, S2 Fig.). Earliest detection occurred at 1 hr for 50 µM DD while 10 µM DD treated cells had significantly increased NO production at 4 hr and 6 hr. Later log phase cells treated with 10 µM and 50 µM DD for 6 hr, showed 1.62 fold and 3.25 fold increases in NO production compared to the solvent control, respectively (Fig 2). Taken together, 10 µM DD was determined to be a suitable sublethal concentration in which cells maintained growth, viability and showed DD responsiveness. This DD concentration is also close to the physiologically relevant concentration that induces reproductive failure in copepods and arrests copepod larval development as reported by previous studies [31, 40].

### Effect of DD on total lipid fatty acid levels in *P*. *tricornutum*

Preliminary experiments using untreated cell cultures characterized the major fatty acids in total lipids by fatty acid methyl ester (FAME) analysis and GC-MS. The major fatty acids peaks were 14:0, 16:0, 18:0, 16:1, 16:2, 16:3, 18:1, 18:2, 20:5, 22:6. These results agreed with previously reported fatty acids in *P*. *tricornutum* [48, 49]. To address the effects of 10 µM DD on fatty acids in total lipids in a 3 and 6 hr measurement period, FAME data were analyzed by determining fold changes in the fatty acids in DD treated cells compared to DMSO solvent control treated cells (Table 1). Internal controls were used to account for losses due to processing of the cells during lipid extraction and preparation of FAMEs Table 1 shows that 10 µM DD treatment for 3 hr resulted in a striking reduction in fatty acids levels. All measured saturated and unsaturated fatty acids significantly decreased except for 18:2 (also decreased but not significantly). The significantly changed fatty acid levels at 3 hr ranged from 0.46–0.69 fold of the DMSO solvent control. In 6 hr treated cells, there was a general trend of increasing fatty acid levels but all remained below the DMSO control levels except for 18:1 (1.12 fold) and 18:2 (1.46 fold).

**Table 1:** Fold changes in saturated and unsaturated fatty acids in 10 µM DD treated cells compared to DMSO solvent control at 3 hr and 6 hr. Data presented for 3 hr time point is representative of two independent experiments; 5–8 biological replicates each for DMSO and DD treatments. Data for 6 hr time point is representative of three independent experiments; 5–8 biological reps each for DMSO and DD treatments; numbers in brackets indicate ±SD; Significance * p< = 0.1 and ** p<0.05 calculated by student’s t-test in comparison to the control.

	Fold change (DD treated cells)/ (DMSO solvent control cells)	
Fatty acids	3 hr	6 hr
Saturated		
14:0	0.68** (0.02)	0.81**(0.12)
16:0	0.69* (0.18)	0.92 (0.09)
18:0	0.57** (0.15)	0.74**(0.11)
Unsaturated		
16:1	0.62* (0.22)	0.87 (0.12)
16:2	0.48** (0.16)	0.84** (0.10)
16:2	0.52** (0.16)	0.73** (0.08)
16:2	0.52** (0.19)	0.82 (0.17)
16:3	0.57** (0.14)	0.80** (0.08)
18:1	0.86 (0.19)	1.12** (0.06)
18:2	0.66* (0.20)	1.46** (0.16)
20:5	0.56* (0.24)	0.82** (0.07)
22:6	0.52** (0.16)	0.81** (0.10)

In general, there was a greater decline in polyunsaturated species than monounsaturated and saturated species indicating a shift of fatty acid pools towards more saturated or monounsaturated species. Fold changes in the sum of saturated and monounsaturated fatty acids (SFAs+MUFAs) were 0.65 and that in PUFAs were 0.55 at 3 hr and 0.94 and 0.87, respectively, at 6 hr in DD treated cells compared to DMSO solvent control (Table 2). A possible reason of decline in certain saturated fatty acids may be their conversion to other unsaturated fatty acids or other secondary metabolites by action of various enzymes in fatty acid synthesis pathway. The observed decline in 18:0 at both time points may be partially linked to the observed increase in 18:1 and 18:2 fatty acids at 6 hr due in part to DD induced increased activity of delta 9 desaturase enzyme. For the observed loss of unsaturated fatty acids, we considered whether the loss may be partially correlated to production of oxylipins. Wichard et al. reported that unsaturated C16s and 20:5 act as substrates for downstream oxylipin production in several PUA-producing species such as *Thalassiosira rotula* [19]. Our attempts using the SPME technique did not detect any volatile compounds produced by DD treated *P*. *tricornutum* cells at 3 hr and 6 hr (S3 Fig). Control DD standard (1–10 µM) dissolved in media alone (no cells) showed the expected peak, EI spectra of which matched that of DD. Our findings are similar to the reported lack of detection of volatiles in sonicated *P*. *tricornutum* cells [20]. However Pohnert et al. reported enzymatic production of non-volatile oxylipins from sonicated *P*. *tricornutum* cells, specifically aldehydic acids 9-ONDE and 12 ODTE from 20:5 precursor fatty acid [17]. Further studies, perhaps using methods more sensitive than SPME, are needed to determine whether DD induced *P*. *tricornutum* cells produce volatile or non-volatile oxylipins, which may partially account for the loss of unsaturated fatty acids.

**Table 2:** Analysis of changes in total saturated and monounsaturated fatty acids compared to that of total polyunsaturated fatty acids in DD treated cells at 3 hr and 6 hr. Data presented for 3 hr time point is representative of two independent experiments; 5–8 biological replicates each for DMSO and DD treatments. Data for 6 hr time point is representative of three independent experiments; 5–8 biological reps each for DMSO and DD treatments; numbers in brackets indicate ±SD; Significance * p< = 0.1 calculated by student’s t-test in comparison to the control.

	Ratio (FA Peak Area / Internal Standard Peak Area)				Fold Change (DD treated cells)/ (DMSO solvent control cells)	
	3 hr		6 hr			
	DMSO (0.1%)	10 µM DD	DMSO (0.1%)	10 µM DD	3 hr	6 hr
Total SFAs+MUFAs	1.46 (0.05)	0.96 (0.24)	1.38 (0.94)	1.29 (0.20)	0.65*	0.94
Total PUFAs	1.62 (0.03)	0.89 (0.32)	1.30 (0.87)	1.13 (0.33)	0.55*	0.87

### ESI-MS/MS analysis of lipid classes in DD and DMSO solvent treated *P*. *tricornutum* cells

To understand how changes in lipid classes may correlate with the observed overall decreases in fatty acid levels, lipid classes were first identified and quantified by comparison to signals for peaks of internal standards added in known amounts during ESI-MS/MS. From the total signal (100%), % of total signal for each lipid class was determined. The data were represented as mol% of each class (as signal of 1nmol of internal standard = signal of 1nmol of analyte of interest) (Table 3). Fold changes in lipid classes were calculated in DD treated cells compared to DMSO solvent control (Table 4).

**Table 3:** Mol % of lipid in each head group class in DMSO solvent and 10 µM DD treated cells at 3 hr and 6 hr. Data represent average of 5 biological replicates for DMSO and DD treated cells and numbers in bracket represent standard deviations; ** p<0.05, * p<0.1 as determined by student’s t-test comparing to the control.

Lipid class	Lipid Mol%							
	DMSO (0.1%) control at 3 hr		10µM DD at 3 hr		DMSO (0.1%) control at 6 hr		10µM DD at 6 hr	
DGDG	13.973	(0.972)	13.640	(0.779)	12.764	(1.000)	13.444	(0.360)
MGDG	43.957	(3.731)	43.139	(3.039)	41.981	(5.410)	40.695	(1.121)
SQDG	2.587	(0.465)	3.150	(0.510)	2.491	(0.430)	2.464	(0.262)
LPC	11.529	(1.301)	12.362	(0.944)	12.932	(4.345)	10.707	(0.644)
PC	21.828	(1.708)	22.821	(2.066)	21.553	(1.018)	26.008**	(0.834)
PG	3.715	(0.145)	2.557**	(0.207)	4.402	(1.009)	4.129	(0.618)
PI	3.774	(0.306)	4.011	(0.333)	4.556	(0.888)	3.566*	(0.277)
LPG	0.850	(0.156)	0.930	(0.449)	1.494	(0.643)	0.839*	(0.162)
LPE	0.106	(0.040)	0.131	(0.023)	0.091	(0.053)	0.120	(0.033)
PE	0.211	(0.084)	0.377**	(0.043)	0.174	(0.072)	0.471*	(0.288)
PS	0.048	(0.030)	0.017*	(0.006)	0.037	(0.021)	0.011**	(0.005)
PA	0.009	(0.005)	0.014	(0.006)	0.014	(0.012)	0.011	(0.005)

Glycolipid classes: monogalactosyldiacylglycerol (MGDG), digalactosyldiacyclglycerol (DGDG) and sulfoquinovosyl diacylglycerol (SQDG). Phospholipid classes: phosphotidylcholine (PC), phopshotidylethanolamine (PE), phosphotidylglycerol (PG), phopsphotidylinositol (PI), phosphatidylserine (PS), phosphatidic acid (PA), lysophophatidylcholine (LPC), lysophosphatidylethanolamine (LPE) and lysophosphatidylglycerol (LPG).

**Table 4:** Fold changes in lipid classes in DD treated *P*. *tricornutum* cells compared to DMSO solvent control. Data represent fold changes calculated from 5 bio reps of each DMSO and DD treated cultures; ** p<0.05; * p<0.1 determined by student’s t-test based on Table 3 in comparison to the control.

Lipid class	Fold change in DD treated cells compared to DMSO solvent control	
	3 hr	6 hr
DGDG	0.98	1.05
MGDG	0.98	0.97
SQDG	1.22	0.99
LPC	1.07	0.83
PC	1.05	1.21**
PG	0.69**	0.94
PI	1.06	0.78*
LPG	1.09	0.56*
LPE	1.24	1.32
PE	1.79**	2.70*
PS	0.36*	0.28**
PA	1.58	0.77

Glycolipid classes: monogalactosyldiacylglycerol (MGDG), digalactosyldiacyclglycerol (DGDG) and sulfoquinovosyl diacylglycerol (SQDG). Phospholipid classes: phosphotidylcholine (PC), phopshotidylethanolamine (PE), phosphotidylglycerol (PG), phopsphotidylinositol (PI), phosphatidylserine (PS), phosphatidic acid (PA), lysophophatidylcholine (LPC), lysophosphatidylethanolamine (LPE) and lysophosphatidylglycerol (LPG).

Table 3 presents the lipid classes identified and their lipid mol%: glycolipid classes monogalactosyldiacylglycerol (MGDG), digalactosyldiacyclglycerol (DGDG) and sulfoquinovosyl diacylglycerol (SQDG), and phospholipid classes phosphotidylcholine (PC), phopshotidylethanolamine (PE), phosphotidylglycerol (PG), phopsphotidylinositol (PI), phosphatidylserine (PS), phosphatidic acid (PA), lysophophatidylcholine (LPC), lysophosphatidylethanolamine (LPE) and lysophosphatidylglycerol (LPG). In the 3 hr DMSO solvent treated cells, MGDG (44% of total measured polar lipids) and DGDG (14%) and PC (22%), and LPC (11.5%) were the most abundant glyco- and phospholipids, respectively. Tables 3 and 4 show that there were no significant changes in chloroplast galactolipids (DGDG, MGDG) and chloroplast glycolipid (SQDG) in response to DD at 3 hr and 6 hr.

In contrast to its minimal effect on chloroplast glycolipids, DD treatment had significant effects on certain classes of phospholipids. At 3 hr, there were significant declines in PG and PS lipids to 0.69 and 0.36 fold, respectively, and a significant 1.8 fold increase in PE. At 6 hr, there were significant increases in PC and PE levels by 1.21 and 2.70 fold, respectively whereas PS, PI and LPG levels declined significantly to 0.28, 0.78 and 0.56 fold (Tables 3 and 4). To our knowledge, this is the first report documenting novel rapid (3 hr, 6 hr) phospholipid changes in response to PUA suggesting early membrane lipid remodeling to aid in adaptation to PUA stress in algae.

We examined the data for possible metabolic relationships between lipid species. The Kennedy pathway for synthesis of PE and PC in mammalian, yeast, plant and algal cells is well studied. Plants and algae can synthesize de novo ethanolamine from serine via soluble decarboxylase. However, an indirect route is known to exist wherein PS can be decarboxylated to form PE [50]. As seen in Table 2, at 3 hr, the decline in PS (0.36 fold) was correlated with a proportionate increase in PE (1.79) fold suggesting a possible role of activated decarboxylases in response to DD. However at 6 hr, the substantial PE increase (2.7 fold) was less correlated with PS decrease (0.28 fold) supporting the involvement of other biosynthetic or catabolic pathways.

### Analysis of effect of DD on lipid molecular species within membrane lipid classes

We next examined lipid classes for their composition of acyl chain(s) referred to as lipid molecular species analysis. This analysis shows the number of carbon atoms and the number of double bonds in the acyl chain(s). As the signals were too low to be accurately assessed, we excluded the very low abundance PS and PA from this analysis. Moreover, our analysis was limited to molecular species which could be clearly defined as either saturated or monounsaturated fatty acid containing (SFA+MUFA) (ie, 30:0, 36:1) or polyunsaturated fatty acid containing (PUFA) (i.e, 5 double bond minimum such as 36:6, 40:10). In the case of glycolipids, the analysis was also restricted to species which were significantly altered (p<0.1) comparing DD and DMSO treated cells. While numerous other molecular species were identified, we did not include species with uncertain acyl chain content in these two categories.

Chloroplast glycolipid classes MGDG, DGDG, and SQDG abundances (mol%) were not appreciably changed comparing DD treated cells and solvent control cells. Lipid molecular species analysis of DGDG showed 2 PUFA species with mixed small changes at 6 hr and effectively there was no trend observed in PUFA content (S1 Table). For MGDG, two SFA+MUFA species were identified and similarly showed little net change. For MGDG, 10 out of 10 defined PUFA containing molecular species (both chains have PUFA) decreased at 6 hr in DD treated cells compared to DMSO treated cells (S1 Table). As MGDG was the most abundant lipid class in both DD and DMSO treated cells (about 41–44 mol%), these correlative data provide an indication that PUFA decreases in MGDG may account for a substantial part of the PUFA reduction observed by the FAME analysis. For SQDG, two SFA+MUFA species (30:1 and 32:1) were identified and significantly increased in DD treated cells (1.19 and 1.36 fold) at 6 and 3 hr respectively (S1 Table).

In phospholipid classes, molecular species analysis was carried out for PC, LPC, PE, LPE in DD treated cells compared to DMSO solvent control (S2–S5 Tables). A summary of SFA+MUFA and PUFA molecular species changes in these lipid classes is presented in Table 5. For analysis of fold changes in SFAs and MUFAs (SFA+MUFAs), the sum of molecular species that were clearly identified as SFA and MUFA (as done above for glycolipids) in DD treated cells were divided by sum of those in DMSO control for both time points. Similarly, for analysis of fold changes in PUFAs, sum of molecular species that were clearly identified as PUFAs in DD treated cells were divided by sum of those in DMSO control.

**Table 5:** Fold changes in lipid molecular species in PE, PC, LPE and LPC lipid classes in DD and DMSO treated cells at 3 hr and 6 hr.

	Fold changes in DD treated cells compared to DMSO solvent control							
	PE		PC		LPC		LPE	
	3hr	6hr	3hr	6hr	3hr	6hr	3hr	6hr
SFAs+MUFAs	2.33	3.35	1.45	1.57	1.41	1.03	1.47	1.76
PUFAs	1.70	2.69	0.99	1.18	0.86	0.69	1.13	1.04

The lipid class PE (mol%) showed a significant increase in both 3 and 6 hr in DD treated cells (1.79, 2.70 fold, respectively) (Table 4). The lipid class PC (mol%) was not appreciably changed in DD treated cells at 3 hr but significantly increased (1.21 fold) at 6 hr (Table 4). Table 5 shows that PE and PC exhibited a larger increase in SFA+MUFA than PUFA molecular species content in DD treated cells compared to the solvent control. The LPC and LPE lipid classes, which had not shown significant changes in levels, also showed larger gains in SFA+MUFA molecular species in DD treated cells compared to PUFA molecular species, which actually decreased in LPC.

As PE and PC lipid classes were significantly increased in abundance in DD treated cells, a summary of significant molecular species changes is included here from S2 and S3 Tables to show the diversity of the observed changes in composition. In this summary, significantly changing molecular species that were identified but were not clear on being in either the SFA+MUFA or PUFA categories are included for completeness. Measured molecular species in PE were C32 to 42 containing at least one double bond (double bonds 1 to 11) except for saturated 32:0 (S2 Table). At 3 and 6 hr of DD treatment, 36:1 increased 10.48 fold and 10.32 fold, respectively and 36:2 increased 5.94 and 5 fold, respectively, compared to solvent control. At 3 hr of DD treatment, 32:1, 32:2, 34:2, 34:3, 36:5, 36:6, 38:6,38:7,38:8, 40:9 and 42:11 increased between 1.5–3.6 fold. At 6 hr of DD treatment, 32:1 and 38:6 increased 3.7 and 8.2 fold and 34:1, 34:2, 36:4,36:5, 38:7,38:8 increased between 1.3–3.1 fold (S2 Table). Measured molecular species in PC ranged from C30 to 44 (double bonds 1 to12) and saturated 30:0 and 32:0 (S3 Table). The latter increased 1.54 fold at 3 hr. At 3 hr of DD treatment, increased abundance species were 32:1(1.18 fold), 34:1 (3.20 fold), 34:2 (1.24 fold), 36:4 (1.41 fold), 38:4 (1.92 fold), 38:6 (2.32 fold), 40:4 (1.31 fold), 40:7 (1.46 fold), 42:3 (1.18 fold) and 44:12 (1.21 fold). Species that declined were 34:3(0.86 fold), 34:4(0.76 fold), 34:5 (0.85 fold), 36:7 (0.76 fold), 38:7 (0.88 fold), 38:8 (0.66 fold), 38:9 (0.56 fold), 40:9 (0.65 fold) and 40:10 (0.86). At 6 hr of DD treatment, increased abundance species were 32:1 (1.28 fold), 34:2 (1.50 fold), 34:1(3.71 fold), 36:3 (1.34 fold), 36:5 (1.17 fold), 38:4(2.45 fold), 38:6 (4.13 fold), 38:7 (1.17 fold), 40:7 (1.67 fold), 40:8 (1.30 fold) and 42:11 (1.13 fold) while declining species were 32:4 (0.83 fold), 34:4 (0.72 fold), 34:5 (0.77 fold), 36:7 (0.76 fold), 38:5(0.28 fold), 38:9(0.44 fold), and 40:9(0.62 fold) (S3 Table).

The PG lipid class showed a significant decline (0.69 fold) at 3 hr in DD treated cells (Table 4). Lipid molecular species showed a larger decline in PUFAs than SFAs + MUFAs at 3 hr whereas at 6 hr there was a small decline in SFAs+MUFAs but no changes in PUFAs (S6 Table). The lipid class LPG (mol%) significantly declined (0.56 fold) at 6 hr (Table 4). In cells treated with DD for 6 hr, LPG lipid class species 16:0 and 16:1 levels decreased (0.57 fold and 0.46 fold, respectively) (S6 Table). PI lipid class showed a significant decline (0.78 fold) at 6hr of DD treatment (Table 4). At this time, PI class 34:3 increased (1.71 fold) and 32:1 decreased (0.77 fold) (S6 Table).

### Effect of DD on sterols in *P*. *tricornutum*

GC-MS analysis of extracted sterols from *P*. *tricornutum* cells showed that Brassicasterol (C-28 sterol, 24-methylcholest-5,22-dien-3ß-ol) was the dominant sterol along with very low amounts of campesterol (24-methylcholest-5-en-ß-ol) in agreement with earlier reports [51, 52]. Campesterol was not analyzed as the signal was too low in some replicates to be quantified. Peak areas of internal standard cholestenol and sterol signals were determined using chemstation software. Relative ratio/proportion of brassicasterol was measured in DMSO solvent control and DD treated cells by dividing peak area of brassicasterol by peak area of internal standard. Fold changes in brassicasterol in DD treated samples were calculated compared to that of DMSO solvent control (Table 6).

**Table 6:** Average relative ratio of brassicasterol in DMSO solvent and DD treated samples and fold changes in brassicasterol in 10µM DD treated cells compared to DMSO solvent control at 3 hr and 6 hr. Data represent average of 5 biological replicates for DD treatment and 4 biological replicates for DMSO solvent, values in bracket represents SD; **p<0.05 determined by student’s t-test compared to solvent control.

	Ratio at 3 hr		Ratio at 6 hr		Fold change (DD treated cells)/ (DMSO solvent control cells)	
	DMSO (0.1%) solvent control	DD 10 µM	DMSO (0.1%) solvent control	DD 10 µM	3 hr	6 hr
Brassicasterol (C-28 sterol)	1.52 (0.03)	1.30 (0.08)	1.55 (0.11)	1.46 (0.14)	0.86**	0.95

Brassicasterol significantly decreased in DD treated cells (0.86 fold) at 3 hr. However at 6 hr, no significant differences were observed.

### Quantification of neutral lipids using fluorescent BODIPY (505/515) dye by flow cytometer

**Fig 3:**
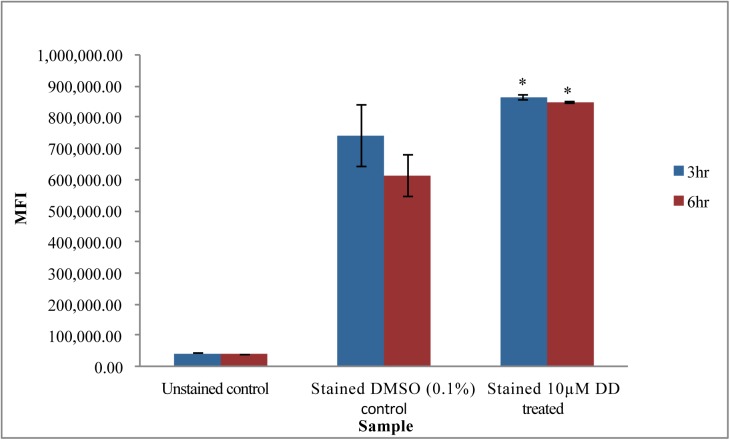
Neutral lipids as quantified by Bodipy dye (505/515). Error bars represent standard error between three biological replicates (* p<0.05 with respect to DMSO solvent control). The data are representative of two independent experiments.

Bodipy (505/515) is a common fluorescent dye used to quantify neutral lipids in various microalgae [46, 53, 54]. DMSO solvent controls and DD treated samples were stained with bodipy and assayed by flow cytometry to quantify neutral lipids. DD treated cells significantly accumulated 1.16 and 1.38 fold neutral lipids compared to DMSO control at 3 and 6 hr, respectively (Fig 3).

### Membrane permeability assays

There is considerable evidence that DD and related short chain lipid aldehydes enter cells through the lipid bilayer parts of the plasma membrane. In studies which produced DD containing liposomes for copepod feeding, DD was stably integrated into liposomes by mixing DD with phospholipids in organic solvent such that DD was loaded into the bilayer part of liposomes, not the aqueous interior [55]. Toxicity assays showed that *Saccharomyces cerevisiae* yeast defective in sterol biosynthesis and known to show increased cell permeability were more sensitive to DD toxicity than wild type yeast [56]. A mammalian unsaturated aldehyde produced by lipid peroxidation, 4-hydroxy-2-nonenal, was characterized in molecular detail for its partitioning and movement across a pure phosphotidylcholine bilayer system [57]. We hypothesized that exposure to sublethal concentration of decadienal produced membrane changes that resulted in decreased subsequent decadienal uptake and increased resistance to higher lethal decadienal concentration. The decadienal induced lipid changes, especially in phospholipids, led us to evaluate changes in membrane permeability. Our approach used cell uptake of three cell dyes with different molecular weights and structures (S4 Fig) as possible indicators of plasma membrane permeability change. The experimental design employed minimal and comparable stain incubation, processing and measurement steps after cells were treated in order to elucidate differences in visible or fluorescent dye levels between DD and solvent treated cells.

#### CFDA-SE dye

**Fig 4:**
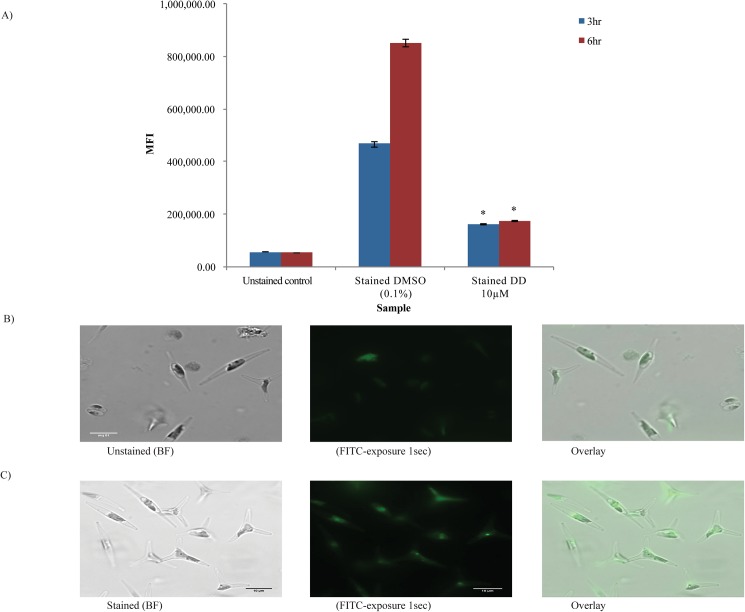
Effects of DD on CFDA-SE staining. A) Analysis of fluorescence of stained cells by flow cytometer (BD Acurri) using CFDA-SE stain B) Bright field image & fluorescence image under FITC (green) of unstained cells (scale bar = 10 µm) and C) cells stained with CFDA-SE dye at 630X magnification. p<0.05 calculated using student’s t-test; n = 3 compared to solvent control. Data are representative of 3 independent experiments.

CFDA-SE (5-(and-6)-Carboxyfluorescein Diacetate, Succinimidyl Ester; MW 557.5) is a non-fluorescent low toxicity dye which generally diffuses well across membranes. Once in the cytoplasm, the two acetate groups are cleaved by esterases to produce the fluorescent form, CFSE. CFSE covalently couples, via its succinimidyl group, to intracellular molecules, particularly to intracellular lysine residues and other amine sources and is stable for a long time inside cells. This dye has been widely used for studying cell lineages and proliferation such as in lymphocytes [58] and also cell divisions in the unicellular alga, Chlorella [59]. Of particular interest, CFSE movement across phospholipid bilayers in liposomes increased in the presence of decadienal and related aldehydes, which the authors attributed to aldehyde perturbations of membrane structure [60]. In our studies, CFSE fluorescence in DD treated cells was 0.34 fold and 0.24 fold the fluorescence in DMSO solvent treated cells at 3 hr and 6 hr, respectively, suggesting decreased cell membrane permeability to CFDA-SE stain in cells pre-treated with DD (Fig 4A). To confirm that CFSE localized in the cells, cells were observed by fluorescence microscopy under FITC filter. Fig 4B shows unstained cells under brightfield and FITC filter and an overlay image. There was very minimal to no background autofluorescence of the cells. Fig 4C shows cells stained with CFSE dye under brightfield, FITC filter (200ms exposure time), and overlay image (800ms exposure time). The images show localization of dye inside the cells with bright fluorescent (green) signal. Lower fluorescence in the DD treated cells is likely due to reduced permeability of dye but other possibilities such as changes in intracellular proteins including esterase levels or efflux mechanisms cannot be excluded. However, the robust esterase mediated conversion of DAF-FM-Diacetate to DAF-FM in DD treated cells (Fig 2) supports that cellular esterases were not limiting and the general intracellular stability of the dye coupled with the short duration of the dye incubation assay supports that efflux or modification of the dye were not major factors.

#### Evans Blue dye

**Fig 5:**
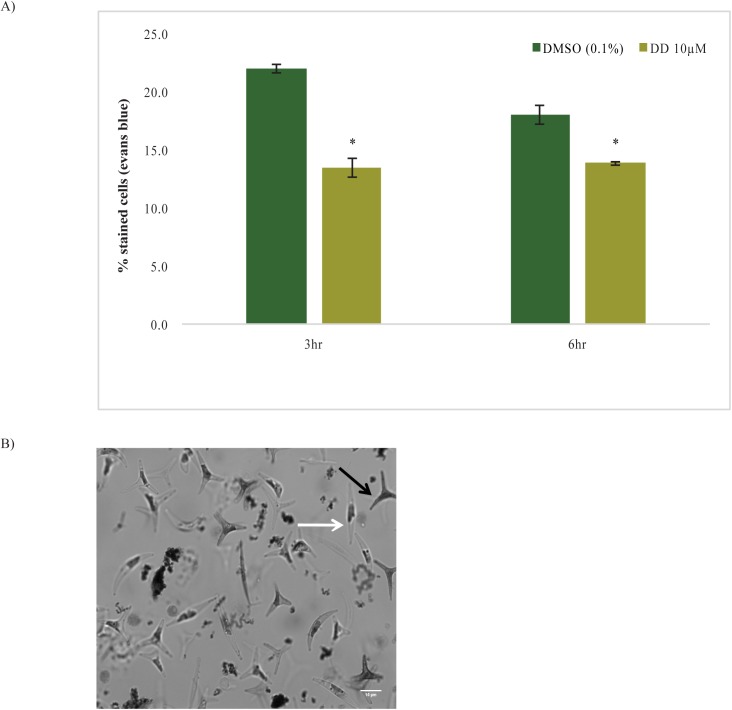
Effects of DD on Evans Blue staining. A) Analysis of % stained cells by cell counting on hemocytometer B) Bright field image (630X) of cells stained with Evans Blue dye (scale bar = 10 µm). p<0.05 calculated using student’s t-test; n = 3 compared to solvent control. Data are representative of 3 independent experiments.

Evans Blue stain (MW 960.8) is a relatively large anionic dye that is excluded by cells with high integrity plasma membranes while it is taken up and imparts a blue color to the interior of cells with damaged or more permeable membranes. While Evans Blue is often referred to as a vital stain which defines living and dead cells, the dye and related Trypan Blue have been extensively used with unicellular organisms including diatoms and in tissues such as muscle fibers to distinguish living cells with varying membrane permeability [47, 61]. In DD treated cells, intracellular Evans Blue staining was significantly lower (13.4% and 13.8%) compared to DMSO treated cells (21.9% and 18%) at 3 hr and 6 hr, respectively (Fig 5A). Evans Blue staining of DMSO treated cells was not a strong function of the DMSO solvent addition as the water control cells showed equivalent staining at 3 hr and 12% staining at 6 hr. Fig 5B shows a gray scale image taken under light microscope of a representative sample wherein gray cells indicate cells stained with Evans Blue compared to non-gray unstained cells. While dye catabolism or efflux could account for the differing staining, we favor the interpretation that in the short time frame of dye incubation and assay, DD treated cells showed reduced Evans Blue staining due to having plasma membranes with reduced PUFA and increased saturated and monounsaturated fatty acid content. The more stable bilayer with this composition may produce fewer transient nanopores, which model studies have shown can admit related Trypan Blue molecules into mammalian cells [62].

#### Hoechst 33342 dye

**Fig 6:**
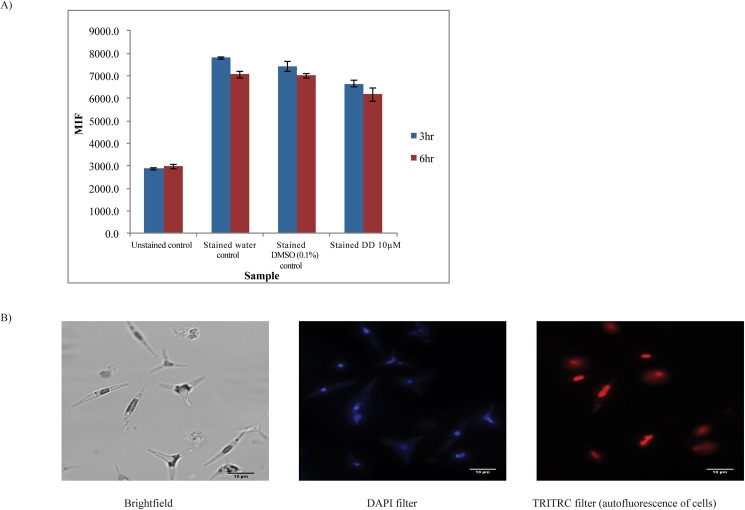
Effects of DD on Hoechst 33342 staining. A) Analysis of fluorescence of stained cells by flow cytometer (BDFACS Aria) using Hoechst 33342 stain B) Bright field image, fluorescence image under DAPI (blue) of stained cells; Autofluorescence of cells (Red) under TRITC filter at 630X magnification (scale bar = 10 µm). p<0.05 calculated using student’s t-test; n = 3 compared to solvent control. Data are representative of 3 independent experiments.

**Fig 7:**
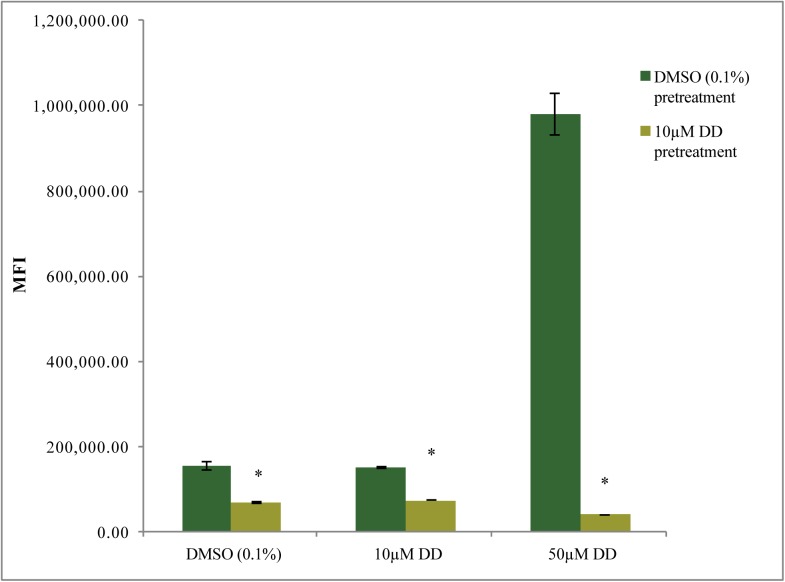
Effects of sublethal DD pretreatment on cell viability after 2 hr sublethal or lethal DD treatments. Cells were pretreated with either DMSO (0.1%) or DD (10 µM) for 3 hr prior to subsequent addition of DMSO (0.1%), 10 µM or 50 µM DD dose for 2 hr. Cell accumulation of Sytox Green fluorescent dye was measured by flow cytometry. MFI is Median Fluorescence Intensity. Error bars represent standard error. Changes in 10 µM pretreated cells were significant with respect to DMSO solvent control pretreated cells, student’s T-test, * p<0.05; n = 3 for DMSO and 10 µM DD data set and n = 2 for 50 µM DD data set.

Hoechst 33342 (MW 561.9) exhibits high partitioning into pure lipid bilayers [63], diffuses across both intact and damaged cell membranes, and exhibits marked increased fluorescence when bound to DNA or partitioned into membranes. It has been used as a probe to measure membrane permeability changes in animal cells [64] and more generally, staining and measuring DNA content in animals, plants and phytoplankton [53, 65, 66]. Unlike CFDA-SE and Evans Blue staining, no significant difference was observed in Hoechst 33342 fluorescence between stained water control, stained DMSO control and stained DD cells at 3 hr and 6 hr (Fig 6A). Fig 7B shows microscope images of cells stained with Hoechst 33342 under DAPI filter (blue) showing the dye was localized to nuclei in cells. DD and DMSO treated cells exhibited comparable intensity of nuclei fluorescence by microscopic observation (data now shown). Under TRITC filter, red autofluorescence of pigments was seen (Fig 6B, right). Thus, the Hoechst 33342 data did not indicate a cell membrane permeability change for this dye in our experimental system. It is well known that different dyes with differing chemical properties and sizes have varying diffusion rates across membranes and that specific bilayer composition differences may or may not impact the rates. Rather, the Hoechst 33342 data supports the conclusion that sublethal decadienal treated cells and control cells were comparable in various processes related to Hoechst 33342 interaction (uptake, localization and DNA binding) within the time period assayed.

#### Membrane integrity assays of cells pretreated with sublethal DD followed by lethal DD concentrations

From the above experiments, cells exposed to sublethal DD concentration underwent shifts in membrane composition and likely reduced cell permeability to several dyes measured 3 and 6 hr after treatment. We hypothesized that uptake of DD may also be lower in these cells, enabling cells to better tolerate high lethal DD doses. To assess this hypothesis, cells were pretreated with either sublethal 10 µM DD or DMSO solvent for 3 hr then subsequently treated with either DMSO solvent, 10 µM DD or 50 µM DD for an additional 2 hr. The lethal DD concentration (50 µM) was chosen based on cell damage and death documented in Fig 1B for this concentration. Sytox Green staining at the end of this period was used an indicator of membrane integrity and cell viability. Fig 7 shows that 10 µM DD pretreated cells had significantly less Sytox Green dye uptake in the DMSO and 10 µM DD 2 hr treatments compared to the DMSO pretreated solvent control (0.45 fold and 0.48 fold, respectively). In the 50 µM DD 2 hr treated cells, 10 µM DD pretreated cells were strikingly less affected by this high DD concentration compared to the DMSO pretreated control cells (0.04 fold Sytox Green level). These data reveal that 10 µM DD pretreatment for 3 hr provided cells with significantly greater membrane integrity and cell viability compared to solvent pretreatment, seen most dramatically in the strong protection afforded against subsequent lethal 50 µM DD treatment.

## Discussion

Oxylipins released by diatoms into the aquatic environment under conditions of grazing, nutrient deprivation, and other adverse conditions have dual properties affecting contacted algal cells. At lower concentration, they may serve as infochemicals which may positively affect their physiology and ecological fitness while at higher concentrations, oxylipins are often toxic leading to reduced cell health or death [13, 34]. A previous study had established that exposure of the diatom *Phaeodactylum tricornutum* to a low level of the PUA oxylipin, decadienal (DD), generated resistance to subsequent higher lethal levels [13]. Our study investigated the hypothesis that sublethal DD levels act by eliciting changes in membrane lipid structure and properties that are protective against a subsequent challenge of lethal DD exposure. An extensive lipidomic analysis revealed sublethal DD treated cells rapidly decreased fatty acids in total lipids and increased levels of PE and PC in a 3 and 6 hr time frame. Concomitantly, these and other membrane lipids exhibited increased content of saturated and monounsaturated acyl chains relative to polyunsaturated acyl chains compared to solvent control cells. Such remodeling of membrane lipids may result in increased membrane stability and, in the plasma membrane, decreased permeability to various external low molecular weight solutes. Evidence of decreased plasma membrane permeability in DD treated cells was obtained, based on reduced uptake of two of three externally applied dyes relative to control cells. Additionally, cells pre-conditioned with a sublethal DD dose for 3 hr then treated with a lethal DD dose for 2 hr exhibited greater membrane integrity as shown by greatly reduced Sytox Green dye uptake compared to the solvent pretreatment comparison group. Taken together, the data are supportive of the hypothesis that membrane remodeling induced by sublethal DD is a key element in the development of cellular resistance to various molecules in the external medium, potentially including PUA such as DD. These findings are important for contributing to understanding of microalgae defense mechanisms. More broadly, the findings may contribute to better understanding of ecological relationships in marine communities and improved cultivation of algae in commercial settings.

Preliminary work established 10 µM as a good sublethal DD concentration based on several criteria. The *P*. *tricornutum* cell cultures growth rate was slower during our lipid sampling conditions times (3 hr and 6 hr of DD treatment) but cell viability was unaffected and significant NO production verified DD responsiveness. Using this culture system, saturated and unsaturated fatty acids from total lipids were analyzed by a relative quantification of FAMES between DD treated and solvent treated cells. All measured saturated and unsaturated fatty acids significantly decreased in cells treated with DD for 3 hr (0.46–0.69 fold the level in solvent treated cells) except for 18:2 (also decreased but not significantly). For example, the two most abundant fatty acids (about 20% and 30% of total fatty acids in glycerolipids, respectively) in *P*. *tricornutum* grown in nutrient replete medium were 16:1 and 20:5 [49]. In our experiments, the levels of 16:1 and 20:5 declined to 0.62 and 0.56 fold at 3 hr, respectively, in DD treated cells compared to control cells. In aggregate, PUFA losses were greater than SFA+MUFA losses resulting in a shift to more saturated and monounsaturated fatty acids in the total lipid pool. The majority of down-regulated lipids recovered partially at 6 hr and 18:1 and 18:2 restored to higher than control cell abundances at 6 hr. The duration and magnitude of altered lipid cellular status is unknown beyond 6 hr and would be valuable to determine.

In diatoms, the main membrane classes are phosopholipids, principally PC in nonplastid membranes and glycolipids, MGDG, DGDG and SQDG, found in chloroplasts. As diatoms have chloroplasts with 4 outer membranes and the outermost one is connected to the ER, the actual composition of the outer membranes in particular are less comparable to higher plant chloroplasts and not known. Analysis of lipid classes identified significantly increased PC and PE levels (1.21 and 2.70 fold respectively in DD treated cells over the solvent control at 6 hr. Other phospholipids surveyed did not change appreciably (LPC, LPE and PA) or decreased in level (PG, PI, and PS, ranging from 0.28 to 0.78 fold). The subcellular location(s) of PC and PE accumulation is unknown, but is more likely to have occurred in nonplastid membranes. The accumulated PC and PE were also unlikely associated with higher cell division in the time frame of the experiment, as the DD treated cells grew more slowly than the solvent control treated cells (Fig 1). We suggest the increased PC and PE may be involved in membrane repair or expansion of intracellular nonplastid membranes. Besides phospholipid head group class abundance differences, PC, PE, LPE and LPC in DD treated cells had acyl chain compositions having more SFA and MUFA and less PUFA than the solvent control. The abundance of chloroplast glycolipid classes did not significantly change but the MGDG data also indicated a shift to less PUFA containing molecular species. Since polyunsaturated fatty acids are more prone to be damaged during oxidative stress, this DD induced shift may help protect fatty acids and membrane integrity in cells from NO and ROS produced under sublethal and higher DD concentration conditions (Fig 2, [13], [36]). In addition, membranes with a higher content of SFA and MUFA relative to MUFA would be expected to exhibit greater membrane stability and possibly decrease its permeability to some solutes.

Under the sublethal DD treatment conditions used in these experiments, our findings suggest a stable overall structure and function of chloroplasts. We observed maintenance of stable levels of the main chloroplast membrane glycolipids. In addition, the modest increase in neutral lipids during the time course of the experiment (Fig 3) is consistent with ongoing photosynthesis shunting carbon to neutral lipids in circumstances of reduced cell growth rate as has been widely observed [49], [67]. Using a redox responsive GFP transgene which was targeted to different cell compartments in *P*. *tricornutum*, it was found that exposure to sublethal DD for 1 to nearly 4 hr resulted in little discernable oxidative stress in chloroplasts measured by this assay [36]. In addition, photosynthetic efficiency remained high in *P*. *tricornutum* wild type cells over several days after sublethal DD addition [41].

Lipid remodeling in *P*. *tricornutum* under sublethal DD conditions had shared and differing characteristics with remodeling observed after sonication which is often used to achieve cell wounding such as during grazing. Specifically, best seen in PUA producer species, sonication induces a dramatic loss of PUFA within 5 min derived from phospholipids while saturated fatty acids remain stable [19]. Lipid changes in response to DD measured at 3 and 6 hr showed substantial losses of both saturated, monounsaturated and polyunsaturated fatty acids. Whether these changes or a subset occur on the minutes time scale remains to be determined but we consider that the accumulation of PE and PC, shift to more saturated fatty acid chains in lipid classes may occur over a longer time frame. The fates of the fatty acids which diminished in the DD treated cells are unknown but we venture may involve catabolic degradation, production of oxylipins, depending on the cellular compartment.

Several studies in microalgae and plants have demonstrated rapid changes in lipid class abundances in response to biotic and abiotic factors. In the marine alga *Emiliania huxleyi* infected with a lytic virus, MGDG and DGDG levels decreased and TAGs increased within 48 hr of viral infection. The latter had more saturated fatty acid chains than TAG from uninfected cells and genes involved in TAG biosynthesis were up-regulated [68]. In Arabidopsis, major phospholipids but not galactolipids decreased when cold acclimated plants (4 ^o^C) were exposed to freezing stress (-8 ^o^C) over 12 hr [45]. Increased abundance of PA and decreased levels of PC suggested activation of phospholipases mainly phospholipase D (PLD). Their experiments with PLD mutant plants confirmed PLD as a key enzyme that was involved in the decline of PC during freezing. Vu et al. [42] showed that leaf wounding in Arabidopsis led to changes in lipidome (decline in many structural/membrane lipids whereas increase in many others) as measured after 45 minutes and 6 hr. The changes were associated with modulation of enzymes in lipid oxidation, acylation, hydrolysis and glycosylation pathways. The observed changes in membrane lipids in DD treated cells may similarly involve the role of PLD, hydrolytic or other lipolytic enzymes and transcriptome changes.

In tests aimed at determining functional effects of DD treatment on plasma membrane properties, it was shown that two of three externally applied dyes, CFDA-SE and Evans Blue, demonstrated decreased intracellular accumulation in DD treated cells relative to control cells. These results are likely due to decreased uptake based on consideration of dye properties and design of the experiment. The third dye, Hoechst 33342, was unaffected by DD treatment. This may be attributed to well known differences in dye and drugs uptake in cells and liposome models depending on structural differences and membrane properties [63]. These experiments provided evidence that sublethal DD treatment results in decreased membrane permeability in support of the hypothesis and may confer resistance to lethal DD concentrations.

In another functional test, cells which had been exposed for 3 hr to 10 µM sublethal DD concentration were far more resistant to a subsequent 50 µM lethal DD challenge dose than control cells. Resistance was revealed by low uptake of Sytox Green dye, a marker for cells with compromised membranes and reduced viability. While these data were consistent with the notion of membrane remodeling during the pretreatment period as a key component providing resistance, a cause and effect relationship remains to be established. The lethal DD dose effect may be attenuated by a combination of plasma membrane permeability changes and induction of intracellular defenses such as aldehyde dehydrogenases [69] and glutathione-S-transferases [70] as used in other organisms to achieve neutralization of internalized reactive lipid aldehydes. Considering diatoms in the natural environment, it is likely that diatom species have evolved multiple mechanisms to tolerate fluctuations in oxylipin concentrations.

More broadly, it would be interesting to evaluate permeability differences to other solutes such as ions, organic small molecules including allelochemicals as there may be cross-protective effects. The duration of membrane permeability differences beyond 6 hr remains to be determined as well. Besides PUA release in regions of grazing, PUA are also released in regions of nutrient deprivation and bloom termination [23]. Overall, this study showed that sublethal DD induced rapid membrane lipid remodeling and evidence for altered plasma membrane permeability, which may have adaptative benefits as cells encounter regions of herbivory and possibly other changes in their environment.

## Supporting information

S1 Fig Measurement of cell viability for early log cells (8-9x10^5^ cells/ml) treated with DMSO and DD dose as indicated for the times indicated (hr). (DOCX)

S2 Fig Effects of DD dose and time on NO accumulation in early log phase cells (8-9x10^5^ cells/ml). (DOCX)

S3 Fig Analysis of volatiles using SPME: Representative GC-MS chromatograms. (DOCX)

S4 Fig Structures of dyes. (DOCX)

S1 Table Mol % of lipid molecular species in DGDD, MGDG and SQDG lipid classes in DMSO solvent (0.1%) control and 10 µM DD treated cells. (DOCX)

S2 Table Mol % of lipid molecular species in PE lipid class in DMSO solvent (0.1%) control and 10 µM DD treated cells. (DOCX)

S3 Table Mol % of lipid molecular species in PC lipid class in DMSO solvent (0.1%) control and 10 µM DD treated cells. (DOCX)

S4 Table Mol % of lipid molecular species in LPE lipid class in DMSO solvent (0.1%) control and 10 µM DD treated cells. (DOCX)

S5 Table Mol % of lipid molecular species in LPC lipid class in DMSO solvent (0.1%) control and 10 µM DD treated cells. (DOCX)

S6 Table Mol % of lipid molecular species in PG, LPG and PI lipid classes in DMSO solvent (0.1%) control and 10 µM DD treated cells. (DOCX)
