# Supplementary material for: Defense related decadienal elicits membrane lipid remodeling in the diatom Phaeodactylum tricornutum
Source: PLoS One. 2017 Jun 5;12(6):e0178761. doi: 10.1371/journal.pone.0178761 (PMC5459460; doi:10.1371/journal.pone.0178761)
Supplement: S1 Fig — (DOCX) [file pone.0178761.s001.docx]

**S1 Fig.** **Measurement of cell viability for early log cells (8-9x10^5^ cells/ml) treated with DMSO and DD dose as indicated for the times indicated (hr).** Cell accumulation of Sytox Green fluorescent dye was measured by flow cytometry. F.U. is Fluorescence units. Error bars represent standard error. Changes with 50 µM DD were found significant with respect to DMSO solvent control, Student’s T-test, * P<0.05; n=3.
