# Supplementary material for: Defense related decadienal elicits membrane lipid remodeling in the diatom Phaeodactylum tricornutum
Source: PLoS One. 2017 Jun 5;12(6):e0178761. doi: 10.1371/journal.pone.0178761 (PMC5459460; doi:10.1371/journal.pone.0178761)
Supplement: S2 Fig — (DOCX) [file pone.0178761.s002.docx]

**S2 Fig. Effects of DD dose and time on NO accumulation in early log phase cells**. NO accumulation in cells (8-9x10^5^ cells/ml) was measured using fluorescent dye DAF-FM diacetate by flow cytometry. F.U. is Fluorescence units. Unstained control and stained DMSO (0.1%) solvent control were used as negative controls. SNP (which is a NO donor) at 500 µM acted as positive control. Error bars represent standard error. *Significance with respect to DMSO solvent control calculated by student’s t-test, P<0.05, n=3.
