# Supplementary material for: Defense related decadienal elicits membrane lipid remodeling in the diatom Phaeodactylum tricornutum
Source: PLoS One. 2017 Jun 5;12(6):e0178761. doi: 10.1371/journal.pone.0178761 (PMC5459460; doi:10.1371/journal.pone.0178761)
Supplement: S3 Fig — (DOCX) [file pone.0178761.s003.docx]

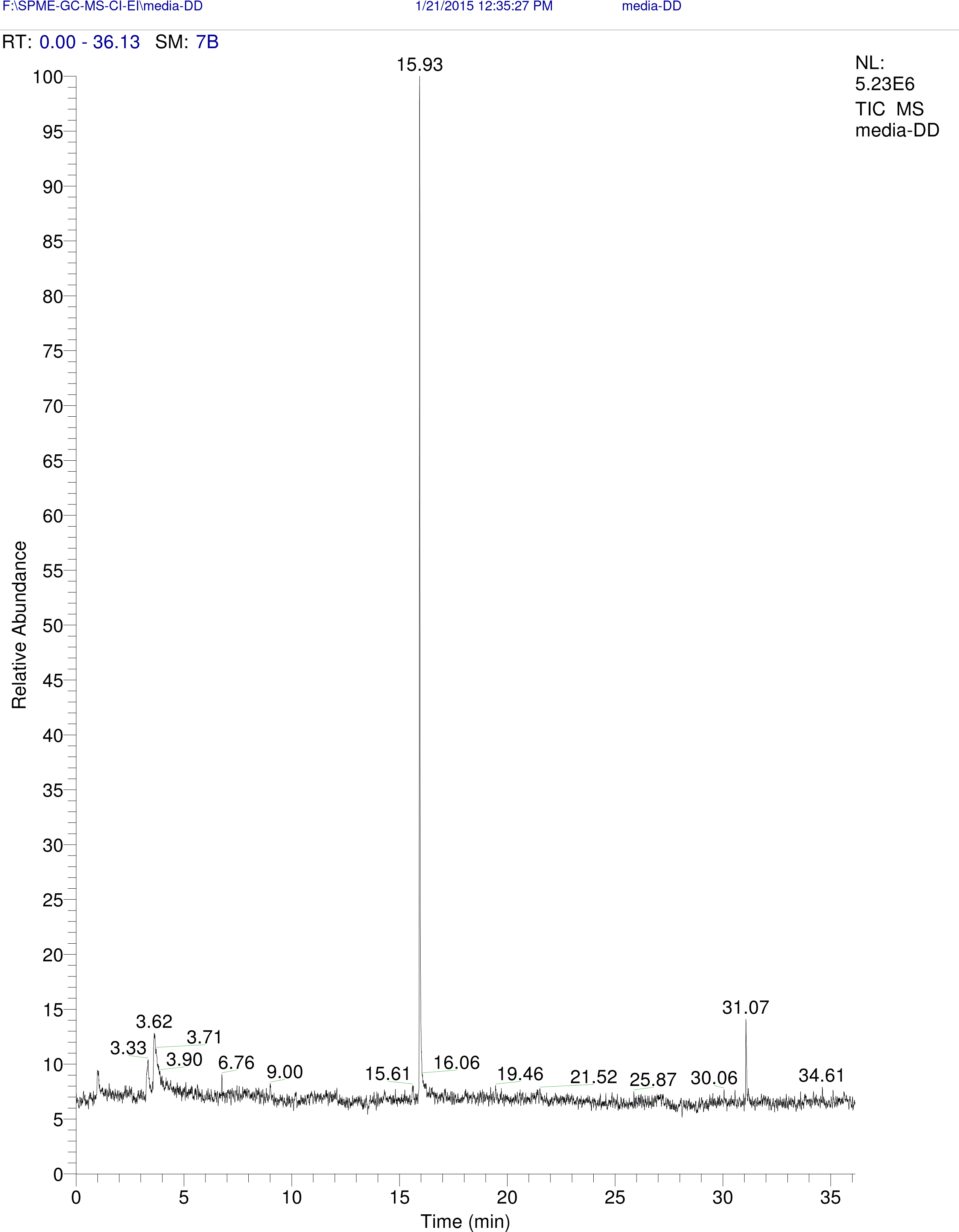


A)


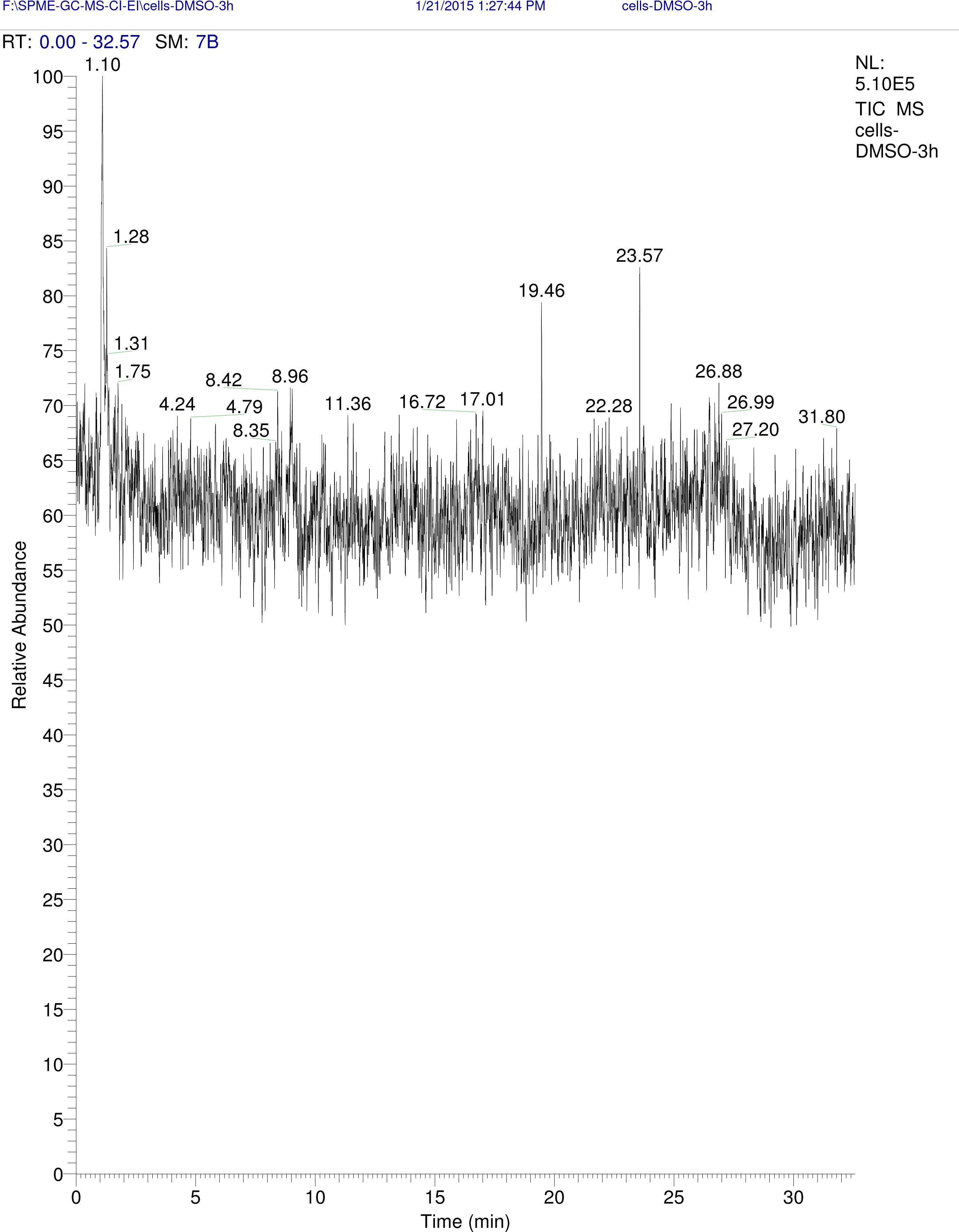


B)

C)


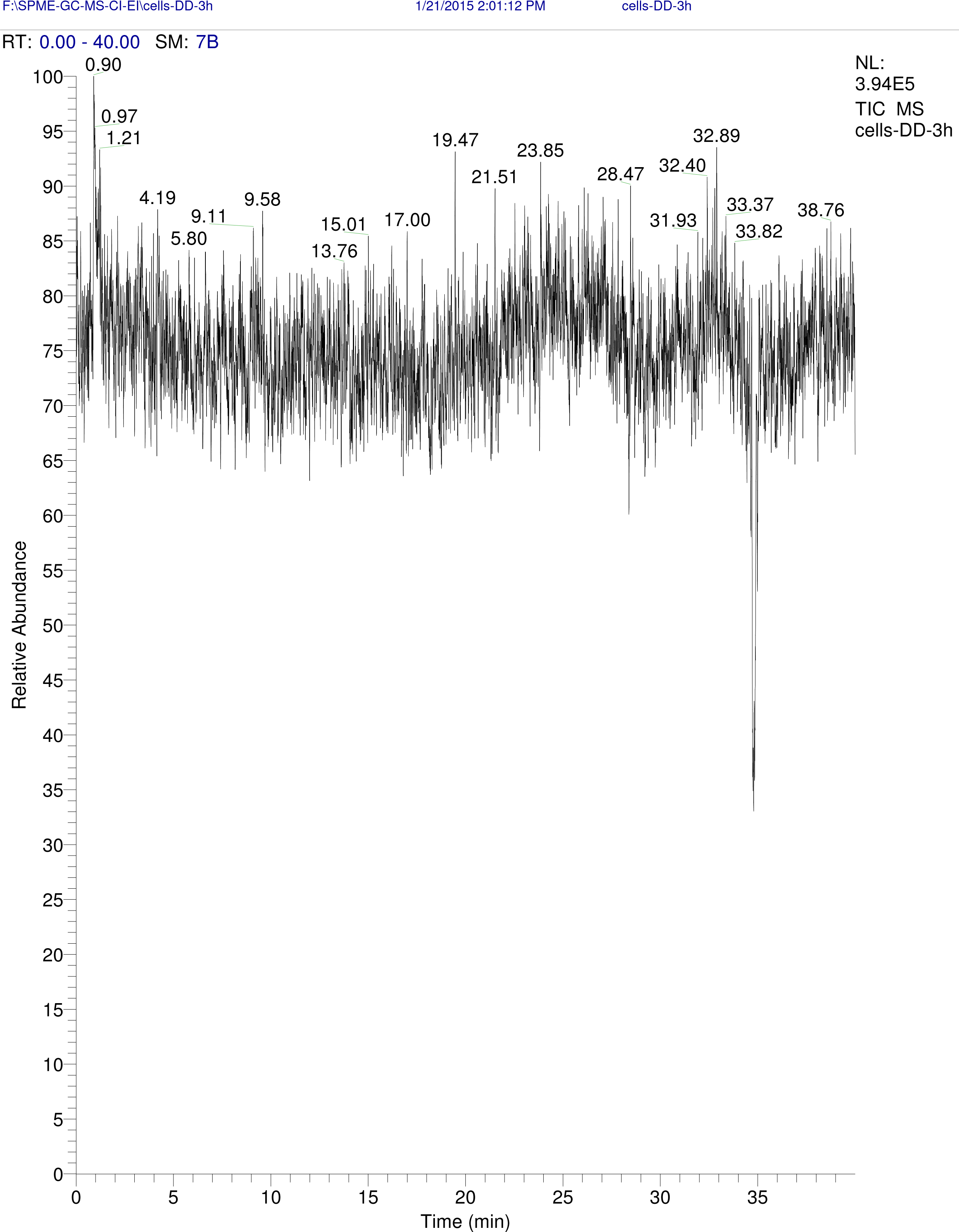


S3 Fig. Analysis of volatiles by SPME: Representative GC-MS Chromatogram of A) 10 µM DD Standard (DD peak seen at time: 15.93, marked by the arrow) B) Cells treated with DMSO (0.1%) for 3 hr C) Cells treated with 10 µM DD for 3 hr.
