# Supplementary material for: Defense related decadienal elicits membrane lipid remodeling in the diatom Phaeodactylum tricornutum
Source: PLoS One. 2017 Jun 5;12(6):e0178761. doi: 10.1371/journal.pone.0178761 (PMC5459460; doi:10.1371/journal.pone.0178761)
Supplement: S4 Fig — (DOCX) [file pone.0178761.s004.docx]

(A)

**
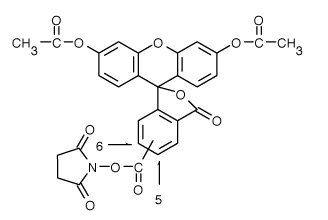
**

(B)


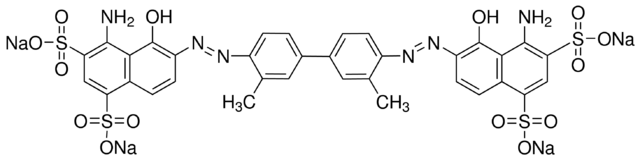


(C)


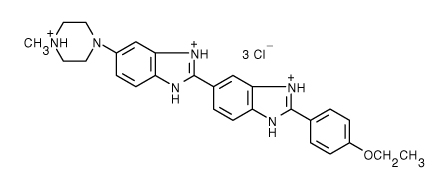


**S3 Fig. Structures of dyes.** (A) CFDA-SE (B) Evans Blue (C) Hoechst 33342 (Structures taken from the database of chemicals called Chemspider: http://www.chemspider.com/)
