# Supplementary material for: Defense related decadienal elicits membrane lipid remodeling in the diatom Phaeodactylum tricornutum
Source: PLoS One. 2017 Jun 5;12(6):e0178761. doi: 10.1371/journal.pone.0178761 (PMC5459460; doi:10.1371/journal.pone.0178761)
Supplement: S1 Table — (DOCX) [file pone.0178761.s005.docx]

**S1 Table. Mol % of lipid molecular species in DGDG, MGDG and SQDG lipid classes in DMSO solvent (0.1%) control and 10 µM DD treated cells**. Data is average of 5 biological replicates and values in bracket represent standard deviation; ** p<0.05, * p<0.1 as determined by student’s t-test compared to solvent control.

|  | **DGDG lipid class** | | | | | | | | |
| --- | --- | --- | --- | --- | --- | --- | --- | --- | --- |
| Lipid Molecular species | Mol% at 3 hr | | | | Mol% at 6 hr | | | | |
|  | DMSO (0.1%) | | 10µM DD | | DMSO (0.1%) | | 10µM DD | | |
| DGDG(32:3) | 0.343 | (0.041) | 0.330 | (0.028) | 0.278 | (0.034) | 0.294 | (0.020) | |
| DGDG(32:2) | 2.010 | (0.301) | 1.664* | (0.144) | 2.090 | (0.130) | 1.801** | (0.114) | |
| DGDG(32:1) | 1.290 | (0.262) | 1.295 | (0.170) | 1.483 | (0.190) | 1.607 | (0.120) | |
| DGDG(34:6) | 0.032 | (0.006) | 0.039* | (0.007) | 0.035 | (0.002) | 0.032 | (0.007) | |
| DGDG(34:5) | 0.080 | (0.007) | 0.074 | (0.010) | 0.073 | (0.016) | 0.070 | (0.008) | |
| DGDG(34:4) | 0.104 | (0.013) | 0.101 | (0.009) | 0.090 | (0.013) | 0.090 | (0.009) | |
| DGDG(34:3) | 0.121 | (0.013) | 0.114 | (0.011) | 0.123 | (0.016) | 0.127 | (0.014) | |
| DGDG(34:2) | 0.098 | (0.023) | 0.086 | (0.012) | 0.093 | (0.013) | 0.095 | (0.016) | |
| DGDG(34:1) | 0.043 | (0.014) | 0.048 | (0.005) | 0.050 | (0.009) | 0.057 | (0.008) | |
| DGDG(36:8) | 0.291 | (0.027) | 0.294 | (0.025) | 0.230 | (0.036) | 0.253 | (0.007) | |
| DGDG(36:7) | 3.399 | (0.373) | 3.160 | (0.157) | 3.134 | (0.503) | 3.041 | (0.094) | |
| DGDG(36:6) | 4.964 | (0.414) | 5.290 | (0.385) | 3.964 | (0.474) | 4.890** | (0.125) | |
| DGDG(36:5) | 0.621 | (0.065) | 0.551 | (0.053) | 0.642 | (0.060) | 0.565* | (0.062) | |
| DGDG(36:4) | 0.051 | (0.011) | 0.056 | (0.018) | 0.048 | (0.013) | 0.052 | (0.009) | |
| DGDG(36:3) | 0.005 | (0.005) | 0.002 | (0.004) | 0.012 | (0.007) | 0.004 | (0.004) | |
| DGDG(36:2) | 0.003 | (0.001) | 0.002 | (0.001) | 0.002 | (0.001) | 0.002 | (0.002) | |
| DGDG(36:1) | 0.010 | (0.008) | 0.009 | (0.003) | 0.013 | (0.006) | 0.008 | (0.005) | |
| DGDG(38:8) | 0.237 | (0.046) | 0.233 | (0.022) | 0.188 | (0.015) | 0.214** | (0.010) | |
| DGDG(38:7) | 0.262 | (0.048) | 0.267 | (0.031) | 0.204 | (0.019) | 0.230 | (0.033) | |
| DGDG(38:6) | 0.000 | (0.000) | 0.016 | (0.017) | 0.004 | (0.007) | 0.004 | (0.006) | |
| DGDG(38:5) | 0.006 | (0.007) | 0.008 | (0.007) | 0.005 | (0.006) | 0.006 | (0.006) | |
| DGDG(38:4) | 0.002 | (0.001) | 0.001 | (0.002) | 0.002 | (0.002) | 0.001 | (0.001) | |
| DGDG(38:3) | 0.002 | (0.001) | 0.000 | (0.001) | 0.001 | (0.001) | 0.001 | (0.001) | |
|  | **MGDG lipid class** | | | | | | | | |
|  | Mol% at 3 hr | | |  | Mol% at 6 hr | | | | |
|  | DMSO (0.1%) | | 10µM DD | | DMSO (0.1%) | | 10µM DD | | |
| MGDG(32:6) | 0.989 | (0.078) | 1.097** | (0.063) | 1.078 | (0.233) | 0.965 | | (0.064) |
| MGDG(32:5) | 3.022 | (0.262) | 3.290 | (0.287) | 2.828 | (0.197) | 2.973 | | (0.187) |
| MGDG(32:4) | 3.904 | (0.309) | 4.369** | (0.348) | 3.353 | (0.384) | 4.005** | | (0.190) |
| MGDG(32:3) | 0.890 | (0.046) | 1.104** | (0.091) | 0.999 | (0.047) | 1.038 | | (0.045) |
| MGDG(32:2) | 2.034 | (0.165) | 1.882 | (0.153) | 2.155 | (0.085) | 1.447** | | (0.051) |
| MGDG(32:1) | 2.031 | (0.335) | 2.577** | (0.229) | 2.238 | (0.242) | 2.037 | | (0.165) |
| MGDG(34:6) | 0.323 | (0.018) | 0.323 | (0.018) | 0.343 | (0.023) | 0.303** | | (0.009) |
| MGDG(34:5) | 0.439 | (0.038) | 0.401 | (0.054) | 0.477 | (0.031) | 0.394** | | (0.029) |
| MGDG(34:4) | 0.312 | (0.025) | 0.291 | (0.028) | 0.410 | (0.011) | 0.290** | | (0.024) |
| MGDG(34:3) | 0.206 | (0.010) | 0.164** | (0.020) | 0.250 | (0.028) | 0.178** | | (0.025) |
| MGDG(34:2) | 0.093 | (0.009) | 0.087 | (0.009) | 0.101 | (0.018) | 0.075** | | (0.007) |
| MGDG(34:1) | 0.104 | (0.007) | 0.111 | (0.007) | 0.105 | (0.014) | 0.097 | | (0.012) |
| MGDG(36:9) | 6.241 | (0.871) | 5.879 | (0.563) | 5.315 | (1.286) | 5.847 | | (0.273) |
| MGDG(36:8) | 17.646 | (2.285) | 16.854 | (1.314) | 16.535 | (3.304) | 16.520 | | (0.721) |
| MGDG(36:7) | 1.388 | (0.145) | 0.972** | (0.085) | 1.217 | (0.233) | 1.040 | | (0.046) |
| MGDG(36:6) | 2.145 | (0.283) | 1.821* | (0.143) | 2.092 | (0.369) | 1.723* | | (0.079) |
| MGDG(36:5) | 0.852 | (0.051) | 0.665** | (0.034) | 0.987 | (0.103) | 0.710** | | (0.060) |
| MGDG(36:4) | 0.031 | (0.010) | 0.021 | (0.007) | 0.047 | (0.027) | 0.029 | | (0.003) |
| MGDG(36:3) | 0.031 | (0.004) | 0.030 | (0.005) | 0.037 | (0.012) | 0.023** | | (0.005) |
| MGDG(36:2) | 0.022 | (0.008) | 0.028 | (0.002) | 0.029 | (0.012) | 0.033 | | (0.004) |
| MGDG(36:1) | 0.055 | (0.012) | 0.048 | (0.010) | 0.071 | (0.008) | 0.056** | | (0.005) |
| MGDG(38:9) | 0.432 | (0.056) | 0.415 | (0.035) | 0.474 | (0.086) | 0.366** | | (0.011) |
| MGDG(38:8) | 0.146 | (0.016) | 0.132 | (0.021) | 0.156 | (0.026) | 0.117** | | (0.004) |
| MGDG(38:7) | 0.204 | (0.028) | 0.150** | (0.011) | 0.208 | (0.023) | 0.130** | | (0.006) |
| MGDG(38:6) | 0.066 | (0.007) | 0.062 | (0.007) | 0.076 | (0.008) | 0.052** | | (0.010) |
| MGDG(38:5) | 0.014 | (0.003) | 0.015 | (0.003) | 0.020 | (0.004) | 0.017 | | (0.004) |
| MGDG(38:4) | 0.006 | (0.004) | 0.032** | (0.017) | 0.004 | (0.004) | 0.008 | | (0.004) |
| MGDG(38:3) | 0.001 | (0.001) | 0.002 | (0.003) | 0.005 | (0.004) | 0.004 | | (0.003) |
| MGDG840.6 | 0.260 | (0.048) | 0.216 | (0.025) | 0.290 | (0.060) | 0.157** | | (0.013) |
| MGDG842.6 | 0.071 | (0.004) | 0.099** | (0.018) | 0.081 | (0.015) | 0.061** | | (0.008) |
|  | **SGDG lipid class** | | | | | | | | |
|  | Mol% at 3 hr | | | | Mol% at 6 hr | | | | |
|  | DMSO (0.1%) | | 10µM DD | | DMSO (0.1%) | | 10µM DD | | |
| SQDG(28:2) | 0.161 | (0.035) | 0.172 | (0.071) | 0.105 | (0.038) | 0.109 | | (0.031) |
| SQDG(30:1) | 0.713 | (0.103) | 0.847 | (0.128) | 0.771 | (0.064) | 0.644** | | (0.048) |
| SQDG(30:2) | 0.411 | (0.063) | 0.559** | (0.099) | 0.400 | (0.071) | 0.411 | | (0.135) |
| SQDG(32:3) | 0.010 | (0.010) | 0.026 | (0.027) | 0.004 | (0.006) | 0.007 | | (0.005) |
| SQDG(32:2) | 0.091 | (0.026) | 0.071 | (0.072) | 0.083 | (0.036) | 0.065 | | (0.044) |
| SQDG(32:1) | 0.394 | (0.234) | 0.630* | (0.119) | 0.461 | (0.071) | 0.316 | | (0.177) |
| SQDG(32:0) | 0.024 | (0.029) | 0.000 | (0.000) | 0.005 | (0.009) | 0.016 | | (0.024) |
| SQDG(34:6) | 0.002 | (0.003) | 0.001 | (0.003) | 0.002 | (0.003) | 0.000 | | (0.000) |
| SQDG(34:5) | 0.031 | (0.025) | 0.050 | (0.011) | 0.044 | (0.035) | 0.028 | | (0.022) |
| SQDG(34:4) | 0.001 | (0.002) | 0.003 | (0.006) | 0.001 | (0.001) | 0.002 | | (0.004) |
| SQDG(34:3) | 0.001 | (0.003) | 0.004 | (0.006) | 0.002 | (0.004) | 0.000 | | (0.001) |
| SQDG(34:2) | 0.000 | (0.000) | 0.002 | (0.005) | 0.001 | (0.002) | 0.001 | | (0.001) |
| SQDG(34:1) | 0.001 | (0.003) | 0.005 | (0.011) | 0.000 | (0.000) | 0.002 | | (0.004) |
| SQDG(36:6) | 0.007 | (0.007) | 0.006 | (0.005) | 0.003 | (0.003) | 0.001 | | (0.002) |
| SQDG(36:5) | 0.317 | (0.020) | 0.347 | (0.055) | 0.278 | (0.075) | 0.225 | | (0.036) |
| SQDG(36:4) | 0.000 | (0.000) | 0.000 | (0.000) | 0.000 | (0.000) | 0.008 | | (0.017) |
| SQDG(36:3) | 0.000 | (0.000) | 0.001 | (0.002) | 0.000 | (0.000) | 0.000 | | (0.000) |
| SQDG(36:2) | 0.000 | (0.000) | 0.000 | (0.000) | 0.001 | (0.003) | 0.000 | | (0.000) |
| SQDG(36:1) | 0.000 | (0.000) | 0.001 | (0.003) | 0.000 | (0.000) | 0.000 | | (0.000) |
