# Supplementary material for: Defense related decadienal elicits membrane lipid remodeling in the diatom Phaeodactylum tricornutum
Source: PLoS One. 2017 Jun 5;12(6):e0178761. doi: 10.1371/journal.pone.0178761 (PMC5459460; doi:10.1371/journal.pone.0178761)
Supplement: S2 Table — (DOCX) [file pone.0178761.s006.docx]

**S2 Table. Mol % of lipid molecular species in PE lipid class in DMSO solvent (0.1%) control and 10 µM DD treated cells**. Data is average of 5 biological replicates and values in bracket represent standard deviation; ** p<0.05, * p<0.1 as determined by student’s t-test compared to solvent control.

|  | PE lipid class | | | | | | | |
| --- | --- | --- | --- | --- | --- | --- | --- | --- |
| Lipid Molecular species | Mol% at 3 hr | | | | Mol% at 6 hr | | | |
|  | DMSO (0.1%) | | 10µM DD | | DMSO (0.1%) | | 10µM DD | |
| **32:1** | 0.005 | (0.002) | 0.009** | (0.001) | 0.004 | (0.002) | 0.013** | (0.006) |
| **32:0** | 0.000 | (0.000) | 0.000 | (0.000) | 0.001 | (0.001) | 0.000 | (0.001) |
| **34:1** | 0.001 | (0.001) | 0.004 | (0.001) | 0.002 | (0.003) | 0.006* | (0.004) |
| **36:1** | 0.000 | (0.000) | 0.003** | (0.001) | 0.000 | (0.000) | 0.003* | (0.003) |
| **32:3** | 0.001 | (0.001) | 0.001 | (0.001) | 0.001 | (0.001) | 0.002 | (0.001) |
| **32:2** | 0.007 | (0.003) | 0.012** | (0.003) | 0.005 | (0.002) | 0.010 | (0.008) |
| **34:4** | 0.002 | (0.002) | 0.004 | (0.001) | 0.002 | (0.001) | 0.005 | (0.004) |
| **34:3** | 0.008 | (0.003) | 0.012** | (0.001) | 0.005 | (0.003) | 0.012 | (0.009) |
| **34:2** | 0.007 | (0.004) | 0.012* | (0.003) | 0.007 | (0.002) | 0.017* | (0.010) |
| **36:6** | 0.017 | (0.008) | 0.031** | (0.004) | 0.011 | (0.007) | 0.032 | (0.020) |
| **36:5** | 0.010 | (0.005) | 0.019** | (0.001) | 0.010 | (0.002) | 0.022* | (0.012) |
| **36:4** | 0.003 | (0.002) | 0.005 | (0.002) | 0.003 | (0.002) | 0.005 | (0.003) |
| **36:3** | 0.001 | (0.001) | 0.002 | (0.002) | 0.002 | (0.001) | 0.001* | (0.001) |
| **36:2** | 0.004 | (0.002) | 0.022** | (0.006) | 0.006 | (0.003) | 0.030* | (0.017) |
| **38:8** | 0.008 | (0.003) | 0.013** | (0.003) | 0.005 | (0.004) | 0.016* | (0.009) |
| **38:7** | 0.030 | (0.010) | 0.047** | (0.006) | 0.021 | (0.009) | 0.060* | (0.035) |
| **38:6** | 0.009 | (0.004) | 0.025** | (0.003) | 0.004 | (0.004) | 0.037** | (0.022) |
| **38:5** | 0.001 | (0.001) | 0.002 | (0.001) | 0.001 | (0.001) | 0.001 | (0.001) |
| **40:10** | 0.039 | (0.015) | 0.053 | (0.005) | 0.032 | (0.015) | 0.072 | (0.049) |
| **40:9** | 0.010 | (0.005) | 0.016** | (0.003) | 0.009 | (0.006) | 0.016 | (0.010) |
| **40:8** | 0.005 | (0.002) | 0.006 | (0.003) | 0.004 | (0.002) | 0.008 | (0.006) |
| **42:11** | 0.042 | (0.019) | 0.078** | (0.008) | 0.039 | (0.015) | 0.102 | (0.064) |
