# Supplementary material for: Defense related decadienal elicits membrane lipid remodeling in the diatom Phaeodactylum tricornutum
Source: PLoS One. 2017 Jun 5;12(6):e0178761. doi: 10.1371/journal.pone.0178761 (PMC5459460; doi:10.1371/journal.pone.0178761)
Supplement: S3 Table — (DOCX) [file pone.0178761.s007.docx]

**S3 Table.** **Mol % of lipid molecular species in PC lipid class in DMSO solvent (0.1%) control and 10 µM DD treated cells**. Data is average of 5 biological replicates and values in bracket represent standard deviation; ** p<0.05, * p<0.1 as determined by student’s t-test compared to solvent control.

|  | PC lipid class | | | | | | | |
| --- | --- | --- | --- | --- | --- | --- | --- | --- |
| Lipid Molecular species | Mol% at 3 hr | | | | Mol% at 6 hr | | | |
|  | DMSO (0.1%) | | 10µM DD | | DMSO (0.1%) | | 10µM DD | |
| 30:1 | 0.190 | (0.033) | 0.182 | (0.012) | 0.200 | (0.019) | 0.194 | (0.009) |
| 30:0 | 0.233 | (0.028) | 0.227 | (0.026) | 0.253 | (0.037) | 0.249 | (0.014) |
| 32:1 | 1.037 | (0.109) | 1.221** | (0.106) | 1.019 | (0.055) | 1.305** | (0.049) |
| 32:0 | 0.187 | (0.024) | 0.288** | (0.045) | 0.219 | (0.079) | 0.266 | (0.021) |
| 34:1 | 0.260 | (0.050) | 0.833** | (0.076) | 0.287 | (0.078) | 1.067** | (0.102) |
| 36:1 | 0.064 | (0.005) | 0.100 | (0.014) | 0.049 | (0.021) | 0.102 | (0.024) |
| 32:4 | 0.129 | (0.036) | 0.127 | (0.023) | 0.122 | (0.012) | 0.101** | (0.009) |
| 32:3 | 0.190 | (0.040) | 0.208 | (0.032) | 0.186 | (0.017) | 0.184 | (0.012) |
| 32:2 | 0.551 | (0.045) | 0.551 | (0.053) | 0.544 | (0.053) | 0.563 | (0.035) |
| 34:5 | 0.521 | (0.039) | 0.441** | (0.041) | 0.589 | (0.078) | 0.452** | (0.036) |
| 34:4 | 0.334 | (0.042) | 0.254** | (0.021) | 0.349 | (0.052) | 0.250** | (0.006) |
| 34:3 | 0.839 | (0.049) | 0.722** | (0.086) | 0.840 | (0.064) | 0.863 | (0.049) |
| 34:2 | 1.044 | (0.099) | 1.292** | (0.121) | 1.124 | (0.157) | 1.688** | (0.089) |
| 36:8 | 0.219 | (0.066) | 0.229 | (0.042) | 0.171 | (0.023) | 0.195 | (0.011) |
| 36:7 | 0.222 | (0.042) | 0.168** | (0.030) | 0.204 | (0.036) | 0.155** | (0.017) |
| 36:6 | 1.335 | (0.090) | 1.314 | (0.132) | 1.290 | (0.098) | 1.239 | (0.125) |
| 36:5 | 3.266 | (0.354) | 3.198 | (0.415) | 3.201 | (0.151) | 3.755** | (0.212) |
| 36:4 | 0.265 | (0.044) | 0.373** | (0.068) | 0.248 | (0.037) | 0.249 | (0.048) |
| 36:3 | 0.257 | (0.031) | 0.305 | (0.043) | 0.253 | (0.064) | 0.339** | (0.022) |
| 36:2 | 0.306 | (0.023) | 0.318 | (0.038) | 0.380 | (0.118) | 0.363 | (0.030) |
| 38:9 | 0.490 | (0.025) | 0.272** | (0.034) | 0.594 | (0.120) | 0.262** | (0.013) |
| 38:8 | 0.573 | (0.075) | 0.378** | (0.050) | 0.593 | (0.134) | 0.517 | (0.052) |
| 38:7 | 2.610 | (0.142) | 2.307** | (0.237) | 2.286 | (0.169) | 2.673** | (0.145) |
| 38:6 | 0.915 | (0.187) | 2.119** | (0.173) | 0.862 | (0.171) | 3.558** | (0.478) |
| 38:5 | 0.212 | (0.025) | 0.208 | (0.017) | 0.191 | (0.052) | 0.053** | (0.080) |
| 38:4 | 0.043 | (0.026) | 0.083** | (0.016) | 0.047 | (0.023) | 0.116** | (0.016) |
| 38:3 | 0.048 | (0.007) | 0.054 | (0.015) | 0.044 | (0.009) | 0.052 | (0.005) |
| 38:2 | 0.064 | (0.007) | 0.068 | (0.010) | 0.054 | (0.019) | 0.067 | (0.012) |
| 40:10 | 2.584 | (0.249) | 2.212** | (0.229) | 2.647 | (0.305) | 2.332 | (0.226) |
| 40:9 | 0.713 | (0.122) | 0.463** | (0.042) | 0.637 | (0.093) | 0.394** | (0.038) |
| 40:8 | 0.471 | (0.030) | 0.501 | (0.043) | 0.407 | (0.031) | 0.530** | (0.030) |
| 40:7 | 0.143 | (0.028) | 0.208** | (0.027) | 0.108 | (0.019) | 0.181** | (0.022) |
| 40:5 | 0.048 | (0.010) | 0.059 | (0.007) | 0.045 | (0.016) | 0.049 | (0.007) |
| 40:4 | 0.031 | (0.002) | 0.041** | (0.007) | 0.044 | (0.014) | 0.039 | (0.013) |
| 40:3 | 0.053 | (0.005) | 0.059 | (0.012) | 0.051 | (0.018) | 0.061 | (0.006) |
| 40:2 | 0.114 | (0.011) | 0.119 | (0.010) | 0.114 | (0.034) | 0.133 | (0.006) |
| 42:11 | 0.916 | (0.064) | 0.966 | (0.104) | 0.943 | (0.045) | 1.065** | (0.052) |
| 42:10 | 0.081 | (0.011) | 0.074 | (0.014) | 0.064 | (0.015) | 0.054 | (0.031) |
| 42:3 | 0.087 | (0.003) | 0.103** | (0.013) | 0.085 | (0.025) | 0.093 | (0.004) |
| 42:2 | 0.089 | (0.016) | 0.077 | (0.020) | 0.115 | (0.060) | 0.106 | (0.016) |
| 44:12 | 0.052 | (0.008) | 0.063** | (0.007) | 0.052 | (0.014) | 0.060 | (0.006) |
| 44:6 | 0.039 | (0.008) | 0.038 | (0.006) | 0.043 | (0.009) | 0.036 | (0.006) |
