# Supplementary material for: Defense related decadienal elicits membrane lipid remodeling in the diatom Phaeodactylum tricornutum
Source: PLoS One. 2017 Jun 5;12(6):e0178761. doi: 10.1371/journal.pone.0178761 (PMC5459460; doi:10.1371/journal.pone.0178761)
Supplement: S4 Table — (DOCX) [file pone.0178761.s008.docx]

**S4 Table.** **Mol % of lipid molecular species in LPE lipid class in DMSO solvent (0.1%) control and 10 µM DD treated cells** Data is average of 5 biological replicates and values in bracket represent standard deviation; ** p<0.05, * p<0.1 as determined by student’s t-test as compared to solvent control.

|  | LPE lipid class | | | | | | | |
| --- | --- | --- | --- | --- | --- | --- | --- | --- |
| Lipid Molecular species | Mol% at 3 hr | | | | Mol% at 6 hr | | | |
|  | DMSO (0.1%) | | 10µM DD | | DMSO (0.1%) | | 10µM DD | |
| 16:1 | 0.007 | (0.005) | 0.016 | (0.009) | 0.006 | (0.005) | 0.013 | (0.012) |
| 16:0 | 0.015 | (0.007) | 0.011 | (0.010) | 0.018 | (0.015) | 0.012 | (0.006) |
| 18:1 | 0.011 | (0.008) | 0.022 | (0.016) | 0.011 | (0.008) | 0.037** | (0.009) |
| Total SFA+MUFAs | **0.033** |  | **0.049** |  | **0.035** |  | **0.062** |  |
| 18:3 | 0.007 | (0.003) | 0.010 | (0.006) | 0.005 | (0.004) | 0.003 | (0.003) |
| 18:2 | 0.040 | (0.022) | 0.048 | (0.007) | 0.029 | (0.014) | 0.035 | (0.007) |
| 22:6 | 0.025 | (0.007) | 0.024 | (0.001) | 0.021 | (0.014) | 0.020 | (0.011) |
| Total PUFAs | **0.073** |  | **0.082** |  | **0.055** |  | **0.058** |  |
