# Supplementary material for: Defense related decadienal elicits membrane lipid remodeling in the diatom Phaeodactylum tricornutum
Source: PLoS One. 2017 Jun 5;12(6):e0178761. doi: 10.1371/journal.pone.0178761 (PMC5459460; doi:10.1371/journal.pone.0178761)
Supplement: S5 Table — (DOCX) [file pone.0178761.s009.docx]

**S5 Table.** **Mol % of lipid molecular species in LPC lipid class in DMSO solvent (0.1%) control and 10 µM DD treated cells**. Data is average of 5 biological replicates and values in bracket represent standard deviations; ** p<0.05, * p<0.1 as determined by student’s t-test compared to solvent control.

|  | LPC lipid class | | | | | | | |
| --- | --- | --- | --- | --- | --- | --- | --- | --- |
| Lipid Molecular species | Mol% at 3 hr | | | | Mol% at 6 hr | | | |
|  | DMSO (0.1%) | | 10µM DD | | DMSO (0.1%) | | 10µM DD | |
| 16:1 | 0.949 | (0.123) | 0.968 | (0.070) | 1.003 | (0.197) | 0.773* | (0.054) |
| 16:0 | 2.347 | (0.206) | 2.569 | (0.255) | 2.789 | (1.271) | 1.898 | (0.108) |
| 18:1 | 1.076 | (0.430) | 2.645** | (0.188) | 1.378 | (0.784) | 2.713** | (0.249) |
| 18:0 | 0.097 | (0.006) | 0.099 | (0.021) | 0.122 | (0.056) | 0.047** | (0.015) |
| Total SFA+MUFAs | **4.469** |  | **6.281** |  | **5.292** |  | **5.431** |  |
| 18:3 | 0.532 | (0.055) | 0.398** | (0.039) | 0.646 | (0.175) | 0.356** | (0.033) |
| 18:2 | 2.591 | (0.230) | 2.228** | (0.163) | 2.368 | (0.655) | 2.000 | (0.185) |
| 20:5 | 2.900 | (0.335) | 2.362** | (0.277) | 3.231 | (0.660) | 2.018** | (0.111) |
| 22:6 | 1.037 | (0.078) | 1.092 | (0.098) | 1.396 | (0.799) | 0.902 | (0.076) |
| Total PUFAs | **7.060** |  | **6.080** |  | **7.641** |  | **5.276** |  |
