# Supplementary material for: Defense related decadienal elicits membrane lipid remodeling in the diatom Phaeodactylum tricornutum
Source: PLoS One. 2017 Jun 5;12(6):e0178761. doi: 10.1371/journal.pone.0178761 (PMC5459460; doi:10.1371/journal.pone.0178761)
Supplement: S6 Table — (DOCX) [file pone.0178761.s010.docx]

**S6 Table.** **Mol % of lipid molecular species in PG, LPG and PI lipid classes in DMSO solvent (0.1%) control and 10 µM DD treated cells**. Data is average of 5 biological replicates and values in bracket represent standard deviations; ** p<0.05, * p<0.1 as determined by student’s t-test compared to solvent control.

| Lipid Molecular species | **PG lipid class** | | | | | | | |
| --- | --- | --- | --- | --- | --- | --- | --- | --- |
|  | Mol% at 3 hr | | | | Mol% at 6 hr | | | |
|  | DMSO (0.1%) | | 10µM DD | | DMSO (0.1%) | | 10µM DD | |
| **(30:1)** | 0.062 | (0.006) | 0.039** | (0.007) | 0.064 | (0.008) | 0.049** | (0.006) |
| **(30:0)** | 0.072 | (0.007) | 0.049** | (0.007) | 0.088 | (0.025) | 0.063* | (0.008) |
| **(32:1)** | 1.455 | (0.114) | 0.896** | (0.069) | 1.885 | (0.450) | 1.492 | (0.249) |
| **(32:0)** | 0.089 | (0.020) | 0.265** | (0.023) | 0.123 | (0.048) | 0.298** | (0.047) |
| **(34:1)** | 0.075 | (0.006) | 0.059** | (0.010) | 0.098 | (0.031) | 0.087 | (0.017) |
| **(34:0)** | 0.010 | (0.006) | 0.011 | (0.002) | 0.010 | (0.004) | 0.014 | (0.003) |
| **(36:1)** | 0.021 | (0.006) | 0.019 | (0.007) | 0.031 | (0.016) | 0.031 | (0.007) |
| **Total SFA+MUFAs** | **1.784** |  | **1.339** |  | **2.300** |  | **2.034** |  |
| **(34:4)** | 0.002 | (0.001) | 0.001 | (0.001) | 0.004 | (0.002) | 0.003 | (0.001) |
| **(34:3)** | 0.014 | (0.004) | 0.010 | (0.003) | 0.018 | (0.004) | 0.014 | (0.004) |
| **(34:2)** | 0.038 | (0.002) | 0.022** | (0.008) | 0.051 | (0.012) | 0.044 | (0.008) |
| **(36:8)** | 0.071 | (0.006) | 0.051** | (0.007) | 0.096 | (0.040) | 0.065 | (0.008) |
| **(36:6)** | 0.900 | (0.036) | 0.483** | (0.066) | 0.797 | (0.328) | 0.866 | (0.141) |
| **(36:5)** | 0.158 | (0.013) | 0.121** | (0.019) | 0.191 | (0.066) | 0.302** | (0.055) |
| **(36:4)** | 0.009 | (0.004) | 0.007 | (0.003) | 0.012 | (0.005) | 0.012 | (0.005) |
| **(36:3)** | 0.015 | (0.003) | 0.007** | (0.002) | 0.015 | (0.003) | 0.013 | (0.002) |
| **(36:2)** | 0.359 | (0.012) | 0.268** | (0.025) | 0.471 | (0.159) | 0.433 | (0.062) |
| **(38:9)** | 0.267 | (0.010) | 0.180** | (0.023) | 0.331 | (0.113) | 0.246 | (0.033) |
| **(38:8)** | 0.068 | (0.009) | 0.045** | (0.006) | 0.075 | (0.021) | 0.058 | (0.006) |
| **(38:2)** | 0.031 | (0.004) | 0.023** | (0.002) | 0.040 | (0.013) | 0.038 | (0.005) |
| **PUFAs total** | **1.931** |  | **1.218** |  | **2.101** |  | **2.095** |  |
|  | **LPG lipid class** | | | | | | | |
|  | Mol% at 3 hr | | | | Mol% at 6 hr | | | |
|  | DMSO (0.1%) | | 10µM DD | | DMSO (0.1%) | | 10µM DD | |
| **(16:1)** | 0.216 | (0.075) | 0.263 | (0.162) | 0.471 | (0.259) | 0.217* | (0.035) |
| **(16:0)** | 0.385 | (0.118) | 0.396 | (0.195) | 0.679 | (0.236) | 0.389** | (0.091) |
| **(18:1)** | 0.019 | (0.019) | 0.045 | (0.025) | 0.062 | (0.075) | 0.032 | (0.031) |
| **Total SFA+MUFAs** | **0.619** |  | **0.704** |  | **1.212** |  | **0.638** |  |
| **(18:3)** | 0.004 | (0.006) | 0.005 | (0.005) | 0.002 | (0.001) | 0.002 | (0.002) |
| **(18:2)** | 0.010 | (0.015) | 0.019 | (0.021) | 0.002 | (0.004) | 0.012 | (0.014) |
| **PUFAs total** | **0.014** |  | **0.024** |  | **0.004** |  | **0.014** |  |
|  | **PI lipid class** | | | | | | | |
|  | Mol% at 3 hr | | | | Mol% at 6 hr | | | |
|  | DMSO (0.1%) | | 10µM DD | | DMSO (0.1%) | | 10µM DD | |
| **(32:3)** | 0.005 | (0.001) | 0.007 | (0.003) | 0.006 | (0.006) | 0.006 | (0.002) |
| **(32:2)** | 0.058 | (0.006) | 0.062 | (0.005) | 0.071 | (0.015) | 0.059 | (0.006) |
| **(32:1)** | 3.639 | (0.302) | 3.863 | (0.312) | 4.385 | (0.846) | 3.421* | (0.276) |
| **(32:0)** | 0.003 | (0.005) | 0.006 | (0.007) | 0.004 | (0.006) | 0.004 | (0.005) |
| **(34:4)** | 0.001 | (0.001) | 0.002 | (0.001) | 0.001 | (0.001) | 0.002 | (0.001) |
| **(34:3)** | 0.005 | (0.001) | 0.008** | (0.002) | 0.009 | (0.004) | 0.007 | (0.002) |
| **(34:2)** | 0.010 | (0.000) | 0.012 | (0.006) | 0.015 | (0.011) | 0.012 | (0.003) |
| **(34:1)** | 0.024 | (0.005) | 0.023 | (0.004) | 0.030 | (0.009) | 0.025 | (0.006) |
| **(36:6)** | 0.006 | (0.003) | 0.006 | (0.003) | 0.007 | (0.002) | 0.006 | (0.002) |
| **(36:5)** | 0.016 | (0.003) | 0.019 | (0.002) | 0.021 | (0.008) | 0.018 | (0.001) |
| **(36:4)** | 0.000 | (0.001) | 0.000 | (0.000) | 0.001 | (0.001) | 0.001 | (0.002) |
| **(36:3)** | 0.001 | (0.001) | 0.001 | (0.001) | 0.002 | (0.002) | 0.002 | (0.001) |
| **(36:2)** | 0.003 | (0.002) | 0.002 | (0.002) | 0.003 | (0.002) | 0.001 | (0.001) |
| **(36:1)** | 0.002 | (0.001) | 0.001 | (0.001) | 0.002 | (0.001) | 0.001 | (0.001) |
